# Development and validation of a web-based patient decision aid for immunotherapy for patients with metastatic melanoma: study protocol for a multicenter randomized trial

**DOI:** 10.1186/s13063-021-05234-4

**Published:** 2021-04-20

**Authors:** Pia Grabbe, Kathrin M. Gschwendtner, Imad Maatouk, Sophia B. Strobel, Martin Salzmann, Julia Bossert, Wolfgang Eich, Beate Wild, Friedegund Meier, Jessica C. Hassel, Christiane Bieber

**Affiliations:** 1 Department of General Internal Medicine and Psychosomatics, Center for Psychosocial Medicine, Heidelberg University Hospital, Thibautstraße 4, 69115 Heidelberg, Germany; 2 National Center for Tumor Diseases, University Hospital Heidelberg, Im Neuenheimer Feld 460, 69120 Heidelberg, Germany; 3 Faculty of Medicine and University Hospital Cologne, University of Cologne, Köln, Germany; 4 TAKEPART Media + Science, Köln, Germany; 5 National Center for Tumor Diseases, University Hospital Dresden, Fetscherstraße 74, 01307 Dresden, Germany

**Keywords:** Melanoma, Immunotherapy, Patient decision aid, Shared decision-making, Randomized controlled trial

## Abstract

**Background:**

Patients with metastatic melanoma and their physicians are confronted with a complex decision regarding first-line therapy. Risks and benefits vary considerably between various treatment options. With this in mind, we aim to develop and evaluate a patient decision aid (PtDA) to inform patients about the risks and benefits of treatment options, namely, immunotherapy as monotherapy, immunotherapy as combination therapy, and treatment with BRAF/MEK inhibitors. We aim to test whether the use of this PtDA before medical consultation will increase patients’ knowledge of treatment options and thus promote shared decision-making (SDM) and patient decision satisfaction.

**Methods:**

In total, 128 patients with metastatic melanoma from two German cancer centers will be randomized to the intervention group (IG), receiving access to the PtDA before medical consultation, or the control group (CG), receiving treatment as usual (TAU), i.e., medical consultation alone. There will be three major assessment points (before intervention, T_0_; after intervention, T_1_; and 3 months after intervention, T_2_). The main outcome is the patient’s knowledge of their treatment options, measured by a self-developed, piloted multiple-choice test at T_1_. Secondary outcome measures will include the extent of SDM during medical consultation, assessed by Observer OPTION 5, and patient decision satisfaction, assessed by the Satisfaction with Decision Scale (SwD), at T_1_ and T_2_.

**Discussion:**

This trial will assess the effectiveness of a developed PtDA to enhance patient knowledge of treatment options for metastatic melanoma, SDM, and patient decision satisfaction. If the efficacy can be proven, the PtDA will be implemented nationwide in Germany to close a relevant gap in the education and care of patients with metastatic melanoma.

**Trial registration:**

ClinicalTrials.gov NCT04240717. Registered on 27 January 2020

## Introduction

### Background

Immunotherapy with immune checkpoint inhibitors is a promising new treatment option for various types of cancer. On the patient side, the impressive successes of immunotherapy are accompanied by high hopes. However, immunotherapies can also cause life-threatening side effects. These are often underestimated or deemphasized by patients in favor of the expected benefit [1].

For the treatment of metastatic melanoma, three immune checkpoint blockers are now approved as monotherapy or combination therapy. Average survival rates have been gradually increased by these immunotherapies, from 6 to 9 months previously [2] to 2 years or more [3–5]. Combination therapy with ipilimumab, a CTLA4 inhibitor, and nivolumab, a PD1 inhibitor, has been approved since 2016 and has the highest response rates to date of 60%, versus 45% in PD1-directed monotherapy [6–8]. The advantage of combination therapy over monotherapy is less clear in the 5-year survival rate of 36%, versus 29% for monotherapy [9], which complicates the treatment decision for physicians and patients. Moreover, while severe adverse events are seen in only 23% of all patients receiving monotherapy, they occur in 59% of patients receiving combination therapy [9]. Hence, the risks and benefits vary considerably between the possible treatment options [10, 11]. In a recent study on treatment choices, melanoma patients (stages I–IV) and their providers systematically accepted all levels of risk for adverse events in exchange for improved survival rates [12]. This finding suggests that patients make extreme, yet conscious, decisions. However, it is also possible that many patients opt for combination therapy without being sufficiently aware of negative side effects, as patients tend to systematically overestimate the benefits and underestimate the risks of an intervention [13].

In addition to the complex decision between monotherapy and combination therapy, approximately half of patients have to consider yet another treatment option, as targeted therapy with BRAF and MEK inhibitors can also be used in the presence of a BRAF mutation (up to 50% of melanomas) [14–16]. Targeted therapy has a higher response rate but does not offer a long-term benefit comparable to that of checkpoint inhibitors, due to the mechanisms of resistance. As the overall survival rate is the strongest determinant of the treatment decision, patients with melanoma (stages I–IV) and their providers systematically prefer combination immunotherapy over targeted therapy [12]. However, in some clinical situations, e.g., in symptomatic patients, the need for tumor reduction might lead to a recommendation of BRAF/MEK inhibition therapy.

For patients and physicians, this is a very complex decision, especially since the treating physician has only a few clinical parameters on which he or she can base a recommendation, including tumor load, tumor growth kinetics, symptoms [17, 18], and medical history. It is thus a typical preference-sensitive decision situation, where the weighing of benefits and risks largely depends on the values of the patient [19]. An optimal decision can therefore only be made if patients are sufficiently informed and involved in a participatory manner. This is underlined in Germany by the Patients’ Rights Act, which came into force in 2013 and stipulates a right to comprehensive and comprehensible information on all treatments, including all possible alternatives and their various side effects, risks, and chances of success [20]. Additionally, the current S3-German treatment guideline recommends the implementation of shared decision-making (SDM) in melanoma care [21]. However, questionnaire studies have revealed that melanoma patients experience unmet information needs and are only partly satisfied with their involvement in the decision-making process, in which they prefer to take an active part [22, 23]. For physicians, it is very challenging to involve patients according to their need for information and participation, as medical facts are very complex and not easy to communicate. In addition, physicians sometimes misinterpret the views of melanoma patients on decision determinants such as quality of life, fear, and trust in medical opinion [12].

One promising way to support patients and physicians in this complex decision-making process is the implementation of a patient decision aid (PtDA). PtDAs are tools that provide evidence-based information about a disease and its treatment options, including the different benefits and risks and their probabilities [24]. They clarify the decision to be made and take into account the preference-sensitive nature of the decision [24]. In this way, PtDAs empower patients to make well-informed, value-congruent decisions together with their treating physicians. Eventually, this leads to fewer decision-making conflicts and greater long-term satisfaction with the decision [25]. PtDAs are not meant to replace but to supplement medical consultations and can be implemented either before or during medical consultation [24]. They facilitate the concept of SDM, i.e., the exchange of information between physician and patient on an equal footing, which enables a joint decision on therapy [26, 27]. In recent years, SDM has become an increasingly important principle in medicine, particularly in oncology [28], and PtDAs have already been successfully used in various medical contexts [25, 29]. However, not all medical decisions are equally suitable for SDM and the use of PtDAs. Previous research has shown that SDM is particularly effective in long-term therapy decisions with high uncertainty and for chronic and life-threatening diseases [30], such as treatment decisions in patients with metastatic melanomas.

There are multiple ways to implement PtDAs, e.g., in booklets, videotapes, and web-based applications. Due to their systematic structure and their ability to integrate new media, web-based PtDAs go far beyond traditional patient education materials in terms of content and quality [31]. For example, web-based PtDAs allow the integration of videos that have been proven to yield greater user satisfaction in patient education than traditional information pamphlets [32].

A PtDA regarding first-line therapies for metastatic melanoma does not yet exist in German-speaking countries. Within the framework of this study, a web-based, interactive PtDA regarding first-line therapies for metastatic melanoma shall be developed and subsequently implemented and evaluated. The development of a PtDA should help to close a knowledge and supply gap. The development phase has already been completed, and a brief overview of the development process and the resulting PtDA is provided. This study protocol mainly focuses on the evaluation of the PtDA. The contents of this protocol are based on the Recommendations for Interventional Trials (SPIRIT) guidelines. The SPIRIT checklist is included in Additional file 2.

### Trial design

This multicentric, two-armed, parallel, randomized, controlled trial compares an intervention group (IG) with access to the PtDA prior to the medical consultation to a control group (CG) receiving treatment as usual, i.e., medical consultation alone. The study design is adapted to the daily clinical care routine, which implies that all patients with metastatic melanoma will receive a medical consultation before they decide on a treatment option together with their physician. The purpose of the trial is the assessment of the efficacy of the newly developed PtDA with the goal of superiority to treatment as usual.

### Objectives

The study aims to evaluate an interactive web-based PtDA for patients with metastatic melanoma. The PtDA is intended to provide information on all first-line therapies but focuses on immunotherapies, as these are suitable for all patients regardless of BRAF mutation status, achieve statistically better long-term effects, and have a mode of action and application that are presumably relatively difficult for patients to understand [9, 33, 34]. The PtDA was developed in strict accordance with the recommendations of the International Patient Decision Aids Standards (IPDAS) Collaboration Background Document [24]. We aim to evaluate whether patients of the IG, who have used the PtDA, have a higher level of knowledge regarding their treatment options compared to the patients in the CG, who have not had access to the PtDA. Additionally, we want to analyze whether the use of the PtDA supports SDM between patients and physicians, as well as patients’ satisfaction with their treatment decision in the short and long term. We propose the following hypotheses.

### Main hypothesis

#### Hypothesis 1

We hypothesize that after using the PtDA, patients will exhibit a higher level of knowledge of first-line treatment options at T_1_ (i.e., after medical consultation) compared to a CG (no PtDA).

### Key secondary hypotheses

#### Hypothesis 2

We hypothesize that after using the PtDA, patients will exhibit a higher level of decision satisfaction at T_2_ (i.e., approx. 3 months after the intervention) compared to a CG (no PtDA).

#### Hypothesis 3

We hypothesize that after using the PtDA, patients and physicians will exhibit a higher level of SDM during medical consultation compared to a CG (no PtDA).

## Methods/design

This multicenter trial will be conducted in the flow of routine patient care at the National Center for Tumor Diseases (NCT) Heidelberg and at the University Cancer Center (UCC) Dresden, Germany.

### Participants

Patients with metastatic melanoma at stage 4 as well as patients at stage 3 whose tumor is not resectable and who will each receive first-line therapy will be eligible for the study. To participate, they must be older than 18 years and have sufficient knowledge of the German language. Patients with limited legal capacity or impairments in this respect, as well as patients with cognitive or physical impairments that affect the processing of the PtDA (e.g., impaired vision) and patients with severe psychiatric or mental illness, will be excluded from the study.

Patients who meet the inclusion criteria will be consecutively recruited by physicians in the day clinics of the study centers. The physicians will explain the course and purpose of the study and provide written information on the study. Patients willing to participate in the study will be required to give written informed consent (see Additional file 1). Patients’ consent to participate in the study can be withdrawn at any time and without giving a reason. The withdrawal will have no negative impact on the patients’ further treatment and will result in immediate termination of their participation in the study.

### Randomization

After obtaining informed consent, randomization will be carried out by trained research assistants in the two study centers using a centralized web-based tool (Randomizer Software, Institute for Medical Informatics, Statistics and Documentation of the Medical University of Graz, www.randomizer.at). Block randomization is applied to guarantee equal sample sizes in each group in case the planned sample size is not achieved. The block size is specified in the web-based tool by the study coordinator and cannot be seen by physicians enrolling participants or research assistants assigning interventions. The allocation ratio for IG and CG is 1:1. Randomization is stratified by the study center. Due to the nature of this trial, blinding of the patients and physicians is not feasible.

### Intervention arm

#### Development of the PtDA

The intervention consists of a web-based, interactive PtDA that educates patients with metastatic melanoma about their first-line treatment options with a focus on immunotherapies. The PtDA was developed by the research group in 2019. The technical and conceptual implementation was realized in cooperation with TAKEPART Media + Science GmbH. Once the PtDA’s objective had been defined, the development process was divided into five major steps: (1) collecting the latest medical evidence for the treatment options to be presented in the PtDA, (2) developing a concept for the structure of the PtDA, (3) translating the medical content into patient-friendly language and forms of presentation (e.g., video clips), (4) presenting a prototype of the PtDA in two focus groups consisting of patients and experts, and (5) revising the prototype based on feedback from the focus groups. The PtDA development process was closely aligned with the international IPDAS criteria [24]. The resulting website consists of nine pages and provides the following content:

- Information about the structure and objectives of the PtDA
- Information about metastatic melanoma and its course
- Information about the mode of action of immunotherapies and the general treatment course
- Information about the differences between immunotherapy as monotherapy and in combination therapy (response rates, adverse events, quality of life)
- Optional information about targeted therapy (eligible patients, mode of action, treatment course, response rates, adverse events)
- Information about other treatment options (participation in clinical trials, complementary medicine, best supportive care)
- Preparation and advice for the following medical consultation
- Interactive module to clarify the patient’s personal values and treatment preferences

#### Delivery of the intervention

Patients randomized to the IG will be asked to use the PtDA on a tablet shortly before their medical consultation takes place. The processing of the PtDA takes approx. 35 min. Beyond the PtDA, patients of the IG will also receive an educational flyer and a note sheet to record their questions and thoughts in advance of the medical consultation. The educational flyer provides a summary of the most important contents of the PtDA. The differences between monotherapy and combination immunotherapy are presented as a so-called Option Grid [35]. Option Grids are tables comparing treatment options based on frequently asked questions. Previous research has shown that regardless of Internet access, many patients prefer print-based information in comparison with web-based information material [36]. Moreover, evidence regarding the satisfaction and competence of older patients when using web-based information, and tablets in particular, is mixed [37–39]. We assume that paper-based information will be a good supplement to the interactive web-based PtDA, not least because the flyer can be taken along to the medical consultation.

After finishing the PtDA, the patients’ individual treatment preferences assessed in the PtDA will be printed and made available to the attending physician to facilitate dialog between physician and patient during medical consultation.

### Treatment as usual

**Fig. 1:**
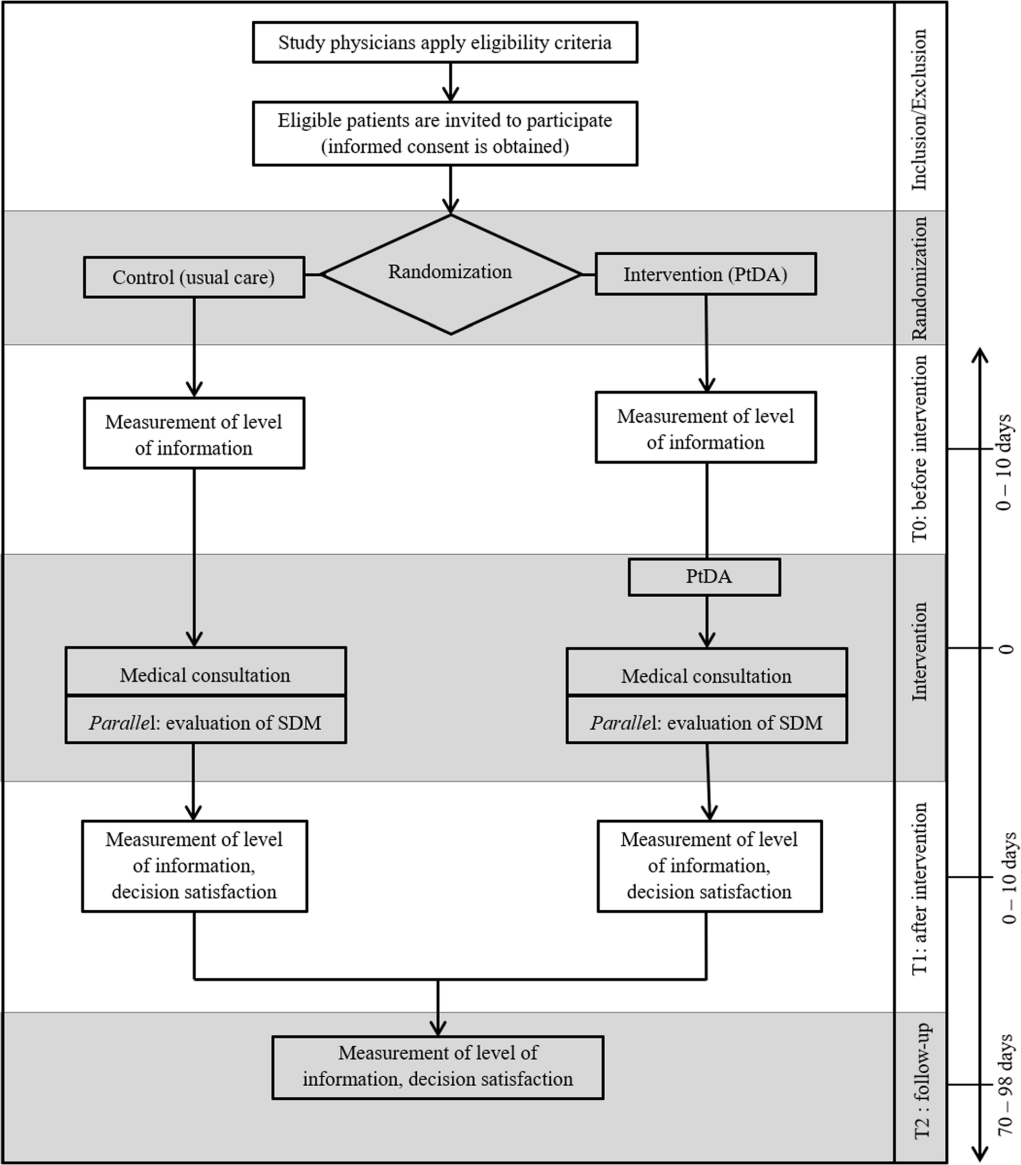
Study design

CG patients will not have access to the PtDA. Instead, they will receive treatment as usual, which consists of a medical consultation on the treatment options available. It is possible that informational material, e.g., as provided by a pharmaceutical company, will be recommended or handed out, as the consultation varies depending on the personal communication style of the treating physician. Medical consultations will be recorded in both the CG and the IG (see Fig. 1). The resulting audio recordings will be transcribed and qualitatively evaluated in terms of SDM (see the “Key secondary outcomes” section).

### Other interventions and concomitant care

As randomization minimizes co-intervention bias, study participants may opt for any additional medical or psychosocial interventions during their participation in the trial (e.g., psycho-oncological therapy or alternative medicine). It is also permitted that they additionally participate in other trials (e.g., when they choose to participate in a drug trial instead of choosing a standard first-line therapy for melanoma). This way, a high ecological validity of study findings is ensured.

### Outcomes

#### Primary outcome

The primary outcome for hypothesis 1 is the patient’s level of knowledge after the intervention, since it is a crucial prerequisite for a common therapeutic decision that patients have understood the risks and benefits of different therapeutic options [25]. Patients’ knowledge will be assessed directly after the intervention (T_1_), in other words, after the medical consultation, and usually on the same day as the medical consultation. Knowledge about first-line treatment options for metastatic melanoma will be assessed with a self-developed, piloted knowledge test (see Additional file 3). This test consists of 10 multiple choice questions in single-selection and multi-selection formats. The scores of the individual tasks are weighed and added up to a total score, which ranges between 0 and 40. Higher scores indicate a higher level of knowledge about treatment options. The reliability of the test, according to an unpublished preliminary study (*N* = 24), is good, with <.8. In addition to T_1_ (primary outcome), patients’ knowledge will also be assessed before the intervention (T_0_) and at the 3-month follow-up (T_2_). T_0_ serves to statistically control for prior knowledge (see Fig. 1).

#### Key secondary outcomes

We will use the German version of the *Satisfaction with Decision Scale* (SwD [40];), extracted from Härter et al. [41], to assess patient decision satisfaction. The scale’s scores range between 6 and 30, with higher scores indicating higher satisfaction with specific health care decisions. As hypothesis 2 refers to decision satisfaction in the further course of the treatment, patient satisfaction at T_2_, i.e., approx. 3 months after the decision, will be analyzed. For further analyses, satisfaction with decisions will be additionally assessed directly after the intervention (T_1_).

Moreover, to test hypothesis 3, a validated German version [42] of the *Observer OPTION-5 scale* [43] will be applied. The Observer OPTION-5 scale is a rater-based observing method to assess SDM regarding a specific health care decision. The scale contains 5 criteria for SDM efforts on the part of the physician that two blinded, independent, trained raters classify on a scale from 0 (*no effort*) to 4 (*exemplary effort*) using audio recordings and transcripts of the medical consultations. The mean value of both raters will be used. The German version of the Observer OPTION-5 has an excellent overall interrater reliability of .82 [42].

#### Further secondary outcomes

As already mentioned, further secondary outcomes are the total score of the knowledge test at T_2_ and SwD scale at T_1_. Moreover, the physicians’ self-rated emotional and cognitive strain during medical consultation will be assessed with two self-developed items at T_1_. On a 5-point Likert scale, physicians will indicate their level of agreement with the following statement: “The consultation challenged me cognitively/emotionally.”

#### Further measurements

To statistically control for possible covariates, the following constructs will be assessed: quality of life, quality of the physician-patient interaction, control preferences of the patient, basic medical information, basic characteristics of the treating physician, and standard sociodemographic variables. Quality of life will be assessed at T_0_ and T_2_ using the *QLQ-C30* [44]. The QLQ-C30 is an established measure in oncology that was developed by the European Organization for Research and Treatment of Cancer (EORTC). It consists of five functional scales (physical, role, cognitive, emotional, and social), three symptom scales (fatigue, pain, and nausea and vomiting), and a global health and quality-of-life scale; it also contains several single-item symptoms. The quality of the physician-patient interaction will be measured with the *Questionnaire on the Quality of Physician-Patient Interaction* (QQPPI [45];), which consists of 14 items that mainly cover the communication skills of the treating physician. Decision control preferences of the patient will be assessed with the German version of the *Control Preferences Scale* (CPS [46];). This scale asks the patient to choose among 5 different roles with increasing levels of autonomy, starting with “I prefer to leave all decisions regarding treatment to my doctor” through “I prefer that my doctor and I share responsibility for deciding which treatment is best for me” and ending with “I prefer to make the decision about which treatment I receive.” For the analyses, the roles will be grouped into three categories, reflecting a passive, a collaborative, and an active approach.

### Data collection and management

**Fig. 2:**
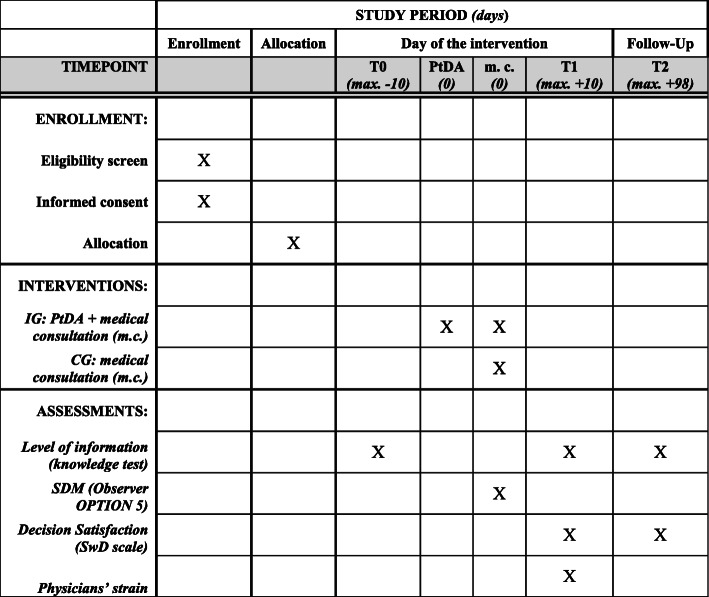
Schedule of enrollment, interventions, and assessments (Standard Protocol Items: Recommendations for Interventional Trials (SPIRIT) figure). Note that the assessment of covariates is not depicted here

The patient’s level of knowledge, decision satisfaction, and quality of life will be assessed by means of paper-based questionnaires, which will be handed out by the trained research assistants or send to the patients per post at the time points displayed in Figs. 1 and 2: T_0_ (0 to 10 days before intervention), T_1_ (0 to 10 days after intervention), and T_2_ (approx. 3 months after intervention). In short, T_0_ and T_1_ usually take place on the same day as the intervention, but can also take place up to 10 days earlier or later if this facilitates their integration into clinical care procedures. To avoid missing values, filled-in questionnaires will be immediately checked for completion by the research assistant. In addition to the self-rating measures, the involvement of the patients in the medical consultation, i.e., SDM, is assessed as an external rating. For this purpose, audio recordings of the medical consultations will be made, transcribed, and evaluated on the basis of the Observer OPTION-5 [43]. In addition, basic medical information on tumor characteristics before and during therapy as well as possible adverse events of the therapy and other physical conditions will be retrieved from medical records.

The close monitoring of the patients by the trained research assistants ensures a high level of adherence to the study protocol. Nevertheless, deviations from the study protocol may occur, for two main reasons: (1) If the research assistants supervising the patients notice excessive psychological strain on the part of the patient, the study procedure will be discontinued or interrupted until the patients’ mental state is stable enough to continue. (2) In case of medical complications, e.g., due to adverse events of the therapy, the follow-up (T_2_) can be rescheduled to an earlier date. In general, the follow-up measurement will take place at slightly different time points, depending on the patient’s staging appointment for a medical checkup. Deviations of 2 weeks before and after the 12th week after the intervention will be tolerated for the follow-up. All other deviations, including those resulting from the abovementioned reasons, will be documented and taken into account in a sensitivity analysis. To audit trial conduct, the study administration (CB and PG) will organize monthly meetings with study physicians and research assistants as well as regular site visits and controls of data quality. This way, corrective actions can be initiated when necessary. Important changes to the study protocol will be communicated to the sponsor as well as to the responsible ethical committee and registered on ClinicalTrials.gov. For organizational reasons, audit measures (except for controls of data quality) will be carried out by a study nurse at the study center in Dresden.

The names of the patients and all other confidential information will be subject to medical confidentiality and the provisions of the Federal Data Protection Act. Third parties will not have access to the original medical records. Each study center will be independently responsible for data collection. All data will be pseudonymized, including the patients’ entries on the PtDA website, which will be transferred to the study centers as an e-mail attachment (PDF document). The questionnaire data collected at the study center in Dresden will be sent by post to Heidelberg, while audio recordings will be transmitted to Heidelberg via the cloud. Data storage, entry, and analysis will be carried out exclusively in Heidelberg. All study data will be treated as strictly confidential and will be evaluated exclusively by the study staff. The data will be stored for 15 years after the completion of the study. The results will be made available to the public by publishing them in a peer-reviewed journal. It will be ensured that no inference can be made about the identity of participants.

### Statistical analysis

#### Sample size

A power analysis was conducted with GPower version 3.1.9.2 with patients’ knowledge about first-line treatment options at T_1_ as the main outcome for the effectiveness of the intervention. According to a Cochrane review, PtDAs improve the level of knowledge with an effect size of *d* = 0.60 [25]. On the basis of a group comparison (*t* test for independent samples) and an *α* error of 5% (two-sided), a case number of *n* = 45 participants per group (total = 90) is sufficient to detect a statistically significant effect of *d* = 0.6 with a power of 80%. To compensate for an assumed drop-out rate of approximately 40%, we plan to include 128 patients in the study.

#### Main analyses

All main analyses are based on the intention-to-treat (ITT) population, which includes all randomized patients except those who withdraw informed consent. Missing values will be assumed to be *missing at random* and replaced by multiple imputation using the *fully conditional specification method* [47]. When imputing multi-item instruments, imputation will be performed at the item level, and the total score will be calculated based on the imputed items [48]. Additionally, as sensitivity analyses, the results obtained for the ITT population will be compared to the results of a sample including only complete cases and to the results of a sample including only complete cases without serious protocol violations (per-protocol analysis).

All analyses will be carried out with SPSS 24. Descriptive analyses will be performed to describe sample characteristics.

Multiple linear regression analyses will be carried out to test the three hypotheses described. To test the main hypothesis, the influence of group membership (IG vs. CG) on the patient’s level of knowledge at T_1_ will be analyzed. The model controls for the following covariates: patient’s level of knowledge at T_0_, age, level of education, and study center.

Secondary outcomes will be analyzed using similar regression models. Additionally, to consider the usefulness of the PtDA from a provider perspective, group differences between physicians educating patients of the IG and the CG will be analyzed in terms of the physicians’ emotional and cognitive strain using the Mann-Whitney *U* test.

## Discussion

In this study, we want to investigate whether an interactive web-based PtDA developed by our research group can effectively support patients with metastatic melanoma in their treatment decision. The main purpose of the PtDA is to empower patients by increasing their knowledge about different treatment options. In studies of patients with various types of cancer, including melanoma, it has been shown that patients have a very high need for information about the disease, treatments, procedures, side effects, and prognosis, especially directly after receiving diagnosis [23, 49–51]. A fulfilled need for information is associated with lower anxiety and depression levels in cancer patients [52]. In the current reality of melanoma care, medical consultation is the main source of information [53]. However, there is usually not enough time for an extensive educational discussion with the physician. Patients therefore have a tendency to inform themselves online, even though available German websites have a medium quality and are difficult to understand, according to reviewer-based assessments [54, 55]. The developed evidence-based PtDA is thus intended to close a gap in clinical care. We assume that a higher level of knowledge also facilitates a greater involvement of patients in the decision-making process in the sense of SDM and increases patients’ decision satisfaction with the course of the selected treatment.

As described above, the evaluation of the PtDA is to be carried out in a multicenter, randomized, controlled study design and comprises several measurement points (T_0_, T_1_, T_2_), since the influence of SDM in general, and presumably of PtDAs in particular, on patient satisfaction can only be expected over the long term [30]. Patients with access to the PtDA before the medical consultation will be compared with patients who receive treatment as usual. The study design is thus adapted to clinical care processes. On the one hand, this guarantees high external validity. On the other hand, this means that the study procedure cannot be extensively standardized. For example, we expect variations in the course of the medical consultation. Sources of this variance are physician-related variables (e.g., professional experience), patient-related variables (e.g., BRAF status), and setting-related variables (e.g., if including the patient in a drug trial is available as an additional option to the standard therapies). Additionally, different progressions after the decision for one treatment option between patients must be considered. It is possible, for example, that a patient may have to discontinue the selected therapy due to severe adverse events, and the follow-up survey T_2_ may therefore have to take place earlier. A further limitation of the design is the lack of the possibility of blinding group membership.

In summary, the project aims to close a relevant gap in the education and care of patients with metastatic melanoma by means of an interactive web-based PtDA. As we have shown, the therapeutic decision for metastatic melanoma is very complex, far-reaching, and partly preference-dependent. Especially with regard to the evaluation of novel immunotherapies, it is to be expected that patients will be overwhelmed by the task of understanding the complex mode of action of these therapies and weighing their various risks and benefits. It is known that a better subjective quality of care in oncological patients can be achieved by a stronger involvement of the patient in therapy decisions [56]. A PtDA empowers the oncological patient to take a more active role in the decision-making process [29] without additional time costs on part of the physician. A PtDA also provides important knowledge, e.g., on how to cope with side effects, which promises better treatment adherence [30]. However, the positive effects of PtDAs depend largely on their conceptual and technical implementation. In the planned evaluation study, proof of efficacy is to be provided under controlled conditions. On this basis, the PtDA can either be further optimized or, if successful, be disseminated throughout Germany.

## Supplementary Information

**Additional file 1.** Informed consent including an English translation.

**Additional file 2.** Standard Protocol Items: Recommendations for Interventional Trials (SPIRIT) 2013 Checklist: recommended items to address in a clinical trial protocol and related documents.

**Additional file 3.** Knowledge test on treatment options regarding metastatic melanoma including an English translation.

**Additional file 4.** Copy of the original ethical approval document including an English translation.

**Additional file 5.** Copy of the original funding document including an English translation.

## Data Availability

The datasets analyzed during the current study are available from the corresponding author on reasonable request. The data will be available after the main publication of them. Any data required to support the protocol can be supplied on request.

## Abbreviations

CG: Control group
CPS: Control Preferences Scale
EORTC: European Organization for Research and Treatment of Cancer
IG: Intervention group
IPDAS: International Patient Decision Aids Standards
ITT: Intention-to-treat
m. c.: Medical consultation
NCT: National Center for Tumor Diseases
PtDA: Patient decision aid
QQPPI: Questionnaire on the Quality of Physician-Patient Interaction (FAPI: Fragebogen zur Arzt-Patienten-Interaktion)
SDM: Shared decision-making
SPSS: Statistical Package for the Social Sciences
SwD: Satisfaction with Decision Scale
TAU: Treatment as usual
UCC: University Cancer Center
QLQ-C30: Quality-of-life Questionnaire
