# Supplementary material for: Development and validation of a web-based patient decision aid for immunotherapy for patients with metastatic melanoma: study protocol for a multicenter randomized trial
Source: Trials. 2021 Apr 20;22:294. doi: 10.1186/s13063-021-05234-4 (PMC8056554; doi:10.1186/s13063-021-05234-4)
Supplement: Supplementary file 1 — Additional file 1. Informed consent including an English translation. [file 13063_2021_5234_MOESM1_ESM.pdf]

UNIVERSITÄTS KLINIKUM HEIDELBERG

NCT NATIONALES CENTRUM FÜR TUMORERKRANKUNGEN HEIDELBERG

getragen von:
Deutsches Krebsforschungszentrum
Universitätsklinikum Heidelberg
Thoraxklinik-Heidelberg
Deutsche Krebshilfe

NCT | Im Neuenheimer Feld 460 | D-69120 Heidelberg

Apl. Prof. Dr. med. Christiane Bieber,
Klinik für Allgemeine Innere Medizin und Psychosomatik,
Zentrum für Psychosoziale Medizin
Thibautstr. 4
D-69115 Heidelberg
Telefon +49 6221 56-38657,
Telefax +49 6221 56-5330
Christiane.Bieber@med.uni-heidelberg.de

PD Dr. med. Jessica Hassel,
Universitätsklinikum Heidelberg,
Hautklinik, Nationales Centrum für Tumorerkrankungen – NCT
Im Neuenheimer Feld 460
D-69120 Heidelberg
Telefon +49 6221 56-38503
Telefax +49 6221 56-4798
Jessica.Hassel@med.uni-heidelberg.de

PD Dr. med. Imad Maatouk
FA für Innere Medizin
Psychoonkologische Ambulanz im NCT
Im Neuenheimer Feld 460
D-69120 Heidelberg
Telefon +49 6221 56-4727
Telefax +49 6221 56-5250
Psychoonkologie@nct-heidelberg.de

Nationales Centrum für Tumorerkrankungen Heidelberg
Im Neuenheimer Feld 460
D-69120 Heidelberg
www.nct-heidelberg.de
info@nct-heidelberg.de

„Partizipative Entscheidungsfindung zur Immuntherapie in der Onkologie – prospektive, randomisiert kontrollierte Studie (PEF-Immun)“

Einwilligungserklärung

Sehr geehrte Patientin, sehr geehrter Patient,
diese Einwilligungserklärung besteht aus zwei Teilen. Der erste Teil bezieht sich auf die Einwilligung in die Studienteilnahme, die für alle Teilnehmer das mehrmalige Ausfüllen von Fragebögen, sowie für Teilnehmer der Kontrollgruppe das Bearbeiten der Entscheidungshilfe beinhaltet. Der zweite Teil bezieht sich auf die Einwilligung in die Verarbeitung Ihrer Daten, welche in der Studie erhoben wurden.
Beachten Sie, dass Sie mit Ihrer Unterschrift sowohl in die Studienteilnahme, als auch in die Datenverarbeitung einwilligen.

1. Einwilligung in die Studienteilnahme

Ich habe die Informationsschrift gelesen und wurde zudem mündlich durch Herrn/Frau ___________________ über das Ziel und den Ablauf der Studie sowie über die Risiken ausführlich und verständlich aufgeklärt. Im Rahmen des Aufklärungsgesprächs hatte ich die Gelegenheit, Fragen zu stellen. Alle meine Fragen wurden zu meiner Zufriedenheit beantwortet. Ich stimme der Teilnahme an der Studie freiwillig zu. Für meine Entscheidung hatte ich ausreichend Zeit. Ein Exemplar der Informationsschrift und der Einwilligungserklärung habe ich erhalten.

2. Einwilligung in die Datenschutzbestimmungen der Studie

Mir ist bekannt, dass bei dieser Studie personenbezogene Daten verarbeitet werden sollen. Die Verarbeitung der Daten erfolgt nach gesetzlichen Bestimmungen und setzt gemäß Art. 6 Abs. 1 lit. a der Datenschutz-Grundverordnung folgende Einwilligungserklärung voraus:

Ich wurde darüber aufgeklärt und stimme freiwillig zu, dass meine in der Studie erhobenen Daten, insbesondere Angaben über meine Gesundheit[1], zu den in der Informationsschrift beschriebenen Zwecken in

---

1 Gemäß Art. 9 Abs. 1 DSGVO handelt es sich bei Gesundheitsdaten um personenbezogene Daten besonderer Kategorie in deren Verarbeitung der Studienteilnehmer ausdrücklich einwilligen muss. Gleiches gilt für Daten, aus denen die

NATIONALES CENTRUM
FÜR TUMORERKRANKUNGEN
HEIDELBERG

getragen von:
Deutsches Krebsforschungszentrum
Universitätsklinikum Heidelberg
Thoraxklinik-Heidelberg
Deutsche Krebshilfe

pseudonymisierter Form aufgezeichnet, ausgewertet und in pseudonymisierter Form zwischen den beiden Studienzentren am Universitätsklinikum Heidelberg und Dresden zum Zwecke der statistischen Auswertung ausgetauscht. Dritte erhalten keinen Einblick in personenbezogene Unterlagen. Bei der Veröffentlichung von Ergebnissen der Studie wird mein Name ebenfalls nicht genannt. Die personenbezogenen Daten werden anonymisiert, sobald dies nach dem Forschungszweck möglich ist. Die Daten werden nach Studienabschluss 15 Jahre aufbewahrt.

Mir ist bekannt, dass diese Einwilligung jederzeit schriftlich oder mündlich ohne Angabe von Gründen widerrufen werden kann, ohne dass mir dadurch Nachteile entstehen. Die Rechtmäßigkeit der bis zum Widerruf erfolgten Datenverarbeitung wird davon nicht berührt. In diesem Fall kann ich entscheiden, ob die von mir erhobenen Daten gelöscht werden sollen oder weiterhin für die Zwecke der Studie verwendet werden dürfen.

Ort, Datum

Name, Vorname der teilnehmenden Person
(in Druckbuchstaben)

Unterschrift der teilnehmenden Person

Aufklärende Person

Der Patient/Proband wurde von mir im Rahmen eines Gesprächs über das Ziel und den Ablauf der Studie sowie über die Risiken aufgeklärt. Ein Exemplar der Informationsschrift und der Einwilligungserklärung habe ich dem Patienten/Probanden ausgehändigt.

Ort, Datum

Name, Vorname der aufklärenden Person
(in Druckbuchstaben)

Unterschrift der aufklärenden Person

---

rassische und ethnische Herkunft , politische Meinungen, religiöse oder weltanschauliche Überzeugungen oder die Gewerkschaftszugehörigkeit hervorgehen, sowie für die Verarbeitung von genetischen Daten, biometrischen Daten zur eindeutigen Identifizierung einer natürlichen Person, Daten zum Sexualleben oder zur sexuellen Orientierung.

UNIVERSITÄTS KLINIKUM HEIDELBERG

NCT NATIONALES CENTRUM FÜR TUMORERKRANKUNGEN HEIDELBERG

getragen von:
Deutsches Krebsforschungszentrum
Universitätsklinikum Heidelberg
Thoraxklinik-Heidelberg
Deutsche Krebshilfe

NCT | Im Neuenheimer Feld 460 | D-69120 Heidelberg

"Shared decision-making on immunotherapy in oncology - prospective randomized controlled trial (PEF-Immun)"

Informed consent

Dear patient,
this form includes two parts. The first part refers to the consent to participate in the study, which involves filling out several questionnaires for all participants and additionally using a Patient Decision Aid (PtDA) for participants in the control group. The second part of the form refers to the consent to analyze your data collected in the study.
Please note that by signing this document you give your consent to both the participation in the study and the use of collected data for statistical analysis.

1. Consent to participate in the study

I read the information document and was also informed verbally by Mr. / Mrs. __________________ about the goal and the course of the study as well as the risks in a detailed and understandable way. I had the opportunity to ask questions during consultation. All my questions were answered to my satisfaction. I voluntarily agree to participate in the study. I had sufficient time for my decision. I have received a copy of the information sheet and the informed consent.

2. Consent to the data protection provisions of the study

I am aware that personal data will be analyzed in this study. The data analysis will be carried out in accordance with legal provisions and, following Art. 6 § 1 lit. a of the "Datenschutzgrundverordnung", requires the following declaration of consent:

I have been informed and voluntarily agree that my data collected in the study, in particular information about my health[1], will pseudonymized for

Apl. Prof. Dr. med. Christiane Bieber,
Klinik für Allgemeine Innere Medizin und Psychosomatik,
Zentrum für Psychosoziale Medizin
Thibautstr. 4
D-69115 Heidelberg
Telefon +49 6221 56-38657,
Telefax +49 6221 56-5330
Christiane.Bieber@med.uni-heidelberg.de

PD Dr. med. Jessica Hassel,
Universitätsklinikum Heidelberg,
Hautklinik, Nationales Centrum für Tumorerkrankungen – NCT
Im Neuenheimer Feld 460
D-69120 Heidelberg
Telefon +49 6221 56-38503
Telefax +49 6221 56-4798
Jessica.Hassel@med.uni-heidelberg.de

PD Dr. med. Imad Maatouk
FA für Innere Medizin
Psychoonkologische Ambulanz im NCT
Im Neuenheimer Feld 460
D-69120 Heidelberg
Telefon +49 6221 56-4727
Telefax +49 6221 56-5250
Psychoonkologie@nct-heidelberg.de

Nationales Centrum für Tumorerkrankungen Heidelberg
Im Neuenheimer Feld 460
D-69120 Heidelberg
www.nct-heidelberg.de
info@nct-heidelberg.de

---

[1]According to Art. 9 § 1 DSGVO, health data are personal data of a special category in whose processing the study participant must expressly consent.

The same applies to data revealing racial or ethnic origin, political opinions, religious or philosophical beliefs or trade union membership, and to the processing of genetic data,

NATIONALES CENTRUM
FÜR TUMORERKRANKUNGEN
HEIDELBERG

getragen von:
Deutsches Krebsforschungszentrum
Universitätsklinikum Heidelberg
Thoraxklinik-Heidelberg
Deutsche Krebshilfe

the purposes described in the information document, analyzed and exchanged in a pseudonymized way between the two study centers at Heidelberg University Hospital and Dresden for the purpose of statistical analysis. Third parties will not be given access to personal documents. My name will also not be mentioned when the results of the study are published. Personal data will be anonymized as soon as this is possible according to the research purpose. The data will be kept for 15 years after completion of the study.

I am aware that this consent can be withdrawn at any time in writing or verbally without giving reasons, without any disadvantages for me. This does not affect the legality of the data processing carried out up to the time of the withdrawal of consent. In this case, I can decide whether the data collected by me should be deleted or whether it may continue to be used for the purposes of the study.

Location, Date

Name, first name of the participating person (in block letters)

Signature of the participating person

Informing person

The patient/proband was informed by me in a conversation about the aim and the course of the study as well as the risks. I gave the patient/test person a copy of the information document and the informed consent.

Location, Date

Name, first name of the provider (in block letters)

Signature of the informing person

biometric data uniquely identifying a natural person, data concerning sexual life or sexual orientation.
