# Supplementary material for: Development and validation of a web-based patient decision aid for immunotherapy for patients with metastatic melanoma: study protocol for a multicenter randomized trial
Source: Trials. 2021 Apr 20;22:294. doi: 10.1186/s13063-021-05234-4 (PMC8056554; doi:10.1186/s13063-021-05234-4)
Supplement: Supplementary file 3 — Additional file 3. Knowledge test on treatment options regarding metastatic melanoma including an English translation. [file 13063_2021_5234_MOESM3_ESM.pdf]

UniversitätsKlinikum Heidelberg

NCT NATIONALES CENTRUM FÜR TUMORERKRANKUNGEN HEIDELBERG

getragen von:
Deutsches Krebsforschungszentrum
Universitätsklinikum Heidelberg
Thoraxklinik-Heidelberg
Deutsche Krebshilfe

Studiencode + Datum (*auszufüllen von der Study Nurse*)

Wissenstest

Der nachfolgende Wissenstest soll herausfinden, wie gut Patientinnen und Patienten mit metastasiertem Melanom über ihre Therapieoptionen informiert sind. Das Ziel ist nicht, Ihre persönliche Leistung zu bewerten, sondern langfristig unser Beratungsangebot zu optimieren. **Es ist daher wichtig, dass Sie nicht raten,** sondern ehrlich angeben, wenn Sie etwas nicht wissen.

Die folgenden Fragen sind Multiple-Choice-Fragen. Zunächst stellen wir Ihnen Fragen, bei denen nur eine Antwortoption richtig sein kann. Danach stellen wir Ihnen Fragen, bei denen mehrere Antwortoptionen richtig sein können.

Für alle Fragen gilt: Wenn Sie bereits eine Antwortoption angekreuzt haben und sich dann noch einmal anders entscheiden möchten, bitten wir Sie, Ihre Angaben wie folgt zu korrigieren:

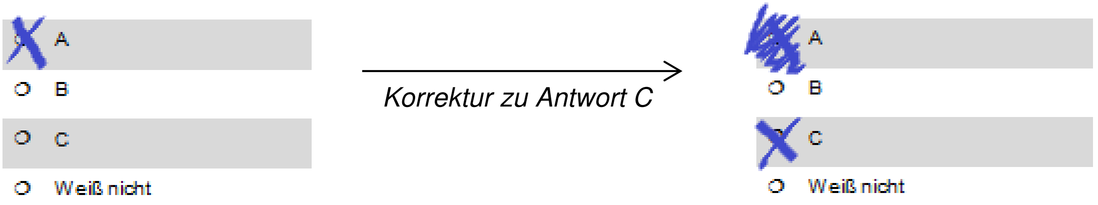

UniversitätsKlinikum Heidelberg

NCT NATIONALES CENTRUM FÜR TUMORERKRANKUNGEN HEIDELBERG

getragen von:
Deutsches Krebsforschungszentrum
Universitätsklinikum Heidelberg
Thoraxklinik-Heidelberg
Deutsche Krebshilfe

Studiencode + Datum (*auszufüllen von der Study Nurse*)

Bei den drei Fragen auf dieser Seite sollen Sie ankreuzen, welche der Antworten richtig ist. **Es ist immer nur eine Antwort richtig.** Wenn Sie nicht wissen, welche Antwort richtig ist, geben Sie bitte „*weiß nicht*“ an.

1. Welche Behandlungsoption ist beim Melanom mit Metastasen und ohne Mutation im BRAF-Gen die erste Wahl?

❍ Bestrahlung

❍ Immuntherapie

❍ Chemotherapie

❍ Weiß nicht

2. Welche Aussage zur BRAF-Mutation (genetische Veränderung im Melanom) ist richtig?

❍ Eine BRAF-Mutation führt zu Lungenmetastasen.

❍ Wenn eine Mutation des BRAF-Gens vorliegt, sind die Immuntherapien nicht wirksam.

❍ Wenn eine Mutation des BRAF-Gens vorliegt, ist eine Therapie mit Tabletten möglich.

❍ Weiß nicht

3. Bei der Immuntherapie mit den sogenannten Checkpointblockern erhalten Patient*innen eine Infusionstherapie. Wie wirkt diese?

❍ Mittels der Infusion werden einzelne Tumorzellen im Blut abgetötet. So wird verhindert, dass Tumorzellen in andere Gewebe eindringen und dort Metastasen entstehen.

❍ Die Substanzen in der Infusion bewirken eine Anregung der körpereigenen Immunabwehr. So wird der Tumor effektiver erkannt und bekämpft.

❍ Die Infusion enthält giftige Substanzen, die den Stoffwechsel der Tumorzellen stören. Dies verhindert die weitere Teilung der Tumorzellen.

UniversitätsKlinikum Heidelberg

NCT

NATIONALES CENTRUM FÜR TUMORERKRANKUNGEN HEIDELBERG

getragen von:
Deutsches Krebsforschungszentrum
Universitätsklinikum Heidelberg
Thoraxklinik-Heidelberg
Deutsche Krebshilfe

Studiencode + Datum (*auszufüllen von der Study Nurse*)

❍ Weiß nicht

Bei den folgenden sieben Fragen zur Immuntherapie sollen Sie ankreuzen, ob die Antwortoptionen richtig oder falsch sind. **Es ist immer mindestens eine Antwort richtig. Es können aber auch mehrere oder alle Aussagen richtig sein.** Wenn Sie nicht wissen, ob eine Antwort richtig oder falsch ist geben Sie bitte „weiß nicht“ an.

Setzen Sie hinter <u>jede</u> Antwortoption <u>ein</u> Kreuz. Zum Beispiel:

Welche Aussage(n) zu den Vorfahrtsregeln ist/sind richtig?

	richtig	*falsch*	*weiß nicht*
Wenn keine Ampeln oder Verkehrszeichen angebracht sind, gilt die Regel rechts vor links.	X	❍	❍
Sind gleichzeitig Ampeln und Verkehrszeichen angebracht, muss man sich nach den Verkehrszeichen richten.	❍	X	❍
Die Rechts-Vor-Links-Regel gilt nicht bei Ausfahrten eines Grundstücks.	❍	❍	X
An einem Stopp-Schild muss man anhalten und Vorfahrt gewähren.	X	❍	❍

4. Welche Aussage(n) zum Ablauf von Immuntherapien ist/sind richtig?

	richtig	*falsch*	*weiß nicht*
Die Wirkstoffe der Immuntherapie werden mittels Infusion verabreicht, die alle 2 Tage erfolgt.	❍	❍	❍
Die Wirkstoffe der Immuntherapie werden mittels Infusion verabreicht, die alle 2-6 Wochen erfolgt.	❍	❍	❍
Für die Infusionsgabe müssen Patient*innen stationär aufgenommen werden und eine Nacht zur Überwachung im Krankenhaus bleiben.	❍	❍	❍
Während der Immuntherapie werden ca. alle 3 Monate Staging-Untersuchungen (Bildgebung mittels CT/MRT) durchgeführt.	❍	❍	❍

UniversitätsKlinikum Heidelberg

NCT NATIONALES CENTRUM FÜR TUMORERKRANKUNGEN HEIDELBERG

getragen von:
Deutsches Krebsforschungszentrum
Universitätsklinikum Heidelberg
Thoraxklinik-Heidelberg
Deutsche Krebshilfe

Studiencode + Datum (*auszufüllen von der Study Nurse*)

5. Welche Aussage(n) zur Mono-Immuntherapie ist/sind richtig?

	richtig	*falsch*	*weiß nicht*
Die Monotherapie führt bei knapp **unter** 50 % der Patient*innen zur Tumorkontrolle (d.h. gleichbleibende oder verringerte Tumorgröße).	❍	❍	❍
Die Monotherapie führt bei knapp **über** 60 % der Patient*innen zur Tumorkontrolle (d.h. gleichbleibende oder verringerte Tumorgröße).	❍	❍	❍
**Mehr** als die Hälfte der Patient*innen mit Monotherapie muss sich wegen starker Nebenwirkungen behandeln lassen.	❍	❍	❍
**Weniger** als ein Viertel der Patient*innen mit Monotherapie muss sich wegen starker Nebenwirkungen behandeln lassen.	❍	❍	❍

6. Welche Aussage(n) zur Kombi-Immuntherapie ist/sind richtig?

	richtig	*falsch*	*weiß nicht*
Die Kombitherapie führt bei knapp **unter** 50% der Patient*innen zur Tumorkontrolle (d.h. gleichbleibende oder verringerte Tumorgröße).	❍	❍	❍
Die Kombitherapie führt bei knapp **über** 60% der Patient*innen zur Tumorkontrolle (d.h. gleichbleibende oder verringerte Tumorgröße).	❍	❍	❍
**Mehr** als die Hälfte der Patient*innen mit Kombitherapie muss sich wegen starker Nebenwirkungen behandeln lassen.	❍	❍	❍
**Weniger** als ein Viertel der Patient*innen mit Kombitherapie muss sich wegen starker Nebenwirkungen behandeln lassen.	❍	❍	❍

UniversitätsKlinikum Heidelberg

NCT NATIONALES CENTRUM FÜR TUMORERKRANKUNGEN HEIDELBERG

getragen von:
Deutsches Krebsforschungszentrum
Universitätsklinikum Heidelberg
Thoraxklinik-Heidelberg
Deutsche Krebshilfe

Studiencode + Datum (*auszufüllen von der Study Nurse*)

7. Welche Aussage(n) zu Nutzen und Risiken von Immuntherapien ist/sind richtig?

	richtig	*falsch*	*weiß nicht*
Unter Kombitherapie ist der Anteil von Patient*innen, bei denen der Tumor kleiner wird, höher als unter Monotherapie.	❍	❍	❍
Unter Kombitherapie ist der Anteil von Patient*innen, bei denen die Erkrankung längerfristig weiter fortschreitet, etwas höher als unter Monotherapie.	❍	❍	❍
Leichte Nebenwirkungen treten bei Mono- und Kombitherapie ungefähr gleich häufig auf.	❍	❍	❍
Bei der Kombitherapie treten schwere Nebenwirkungen häufiger auf als bei der Monotherapie.	❍	❍	❍

8. Welche <u>leichte(n)</u> Nebenwirkung(en) ist/sind typisch für Immuntherapien?

	richtig	*falsch*	*weiß nicht*
Hautausschlag	❍	❍	❍
Müdigkeit	❍	❍	❍
Haarausfall	❍	❍	❍
Gelenkschmerzen	❍	❍	❍

9. Welche <u>schwere(n)</u> Nebenwirkung(en) ist/sind typisch für Immuntherapien?

	richtig	*falsch*	*weiß nicht*
Psychosen (wahnhafte Störung)	❍	❍	❍
Darmentzündungen bzw. Durchfall	❍	❍	❍
Entzündung der Schilddrüse	❍	❍	❍
Entzündung der Leber	❍	❍	❍

UniversitätsKlinikum Heidelberg

NCT NATIONALES CENTRUM FÜR TUMORERKRANKUNGEN HEIDELBERG

getragen von:
Deutsches Krebsforschungszentrum
Universitätsklinikum Heidelberg
Thoraxklinik-Heidelberg
Deutsche Krebshilfe

Studiencode + Datum (*auszufüllen von der Study Nurse*)

10. Welche Aussage(n) zu den Nebenwirkungen von Immuntherapien ist/sind richtig?

	richtig	*falsch*	*weiß nicht*
Zum Erkennen von Nebenwirkungen sind regelmäßige Kontrollen der Blutwerte empfohlen.	❍	❍	❍
Wenn die Immuntherapie wegen Nebenwirkungen abgebrochen werden muss, ist ihre Wirksamkeit nicht verringert.	❍	❍	❍
Bei Abbruch der Immuntherapie verschwinden die Nebenwirkungen immer.	❍	❍	❍
Leichte Nebenwirkungen können symptomatisch behandelt werden (z.B. bei Hautnebenwirkungen mit einer Salbe), und die Immuntherapie kann fortgesetzt werden.	❍	❍	❍

Zuletzt interessiert uns noch, wie gut Sie sich über Ihre Behandlungsoptionen informiert fühlen. Setzen Sie hierzu ein Kreuz auf der Skala.

Beispiel: Wie besorgt sind Sie über den Klimawandel?

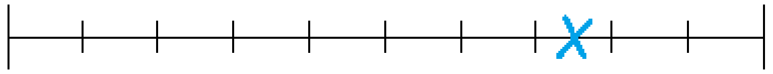

überhaupt nicht maximal besorgt

11. Wie gut fühlen Sie sich momentan über Ihre Behandlungsoptionen informiert?

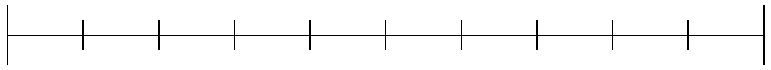

überhaupt nicht maximal informiert

Knowledge test

The following knowledge test is designed to find out how well patients with metastatic melanoma are informed about their treatment options. The aim is not to evaluate your personal performance, but to optimize our consulting services in the long term**. It is therefore important that you do not guess,** but rather honestly state if you do not know something.

The following questions are multiple choice questions. First, we ask you questions where only one answer option can be correct. Then we ask you questions for which several answer options may be correct.

For all questions: If you have already ticked an answer option and would like to change your mind, please correct your details as follows:

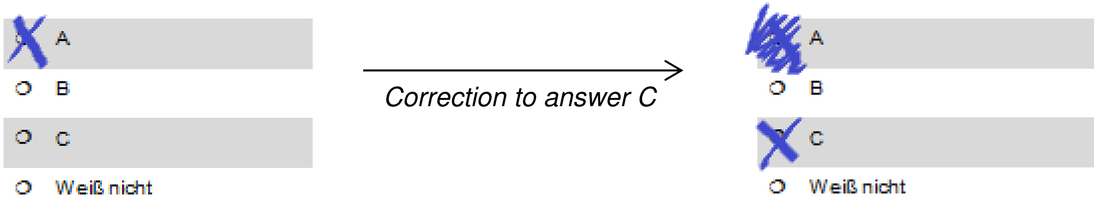

For the three questions on this page you should tick which of the answers is correct. **Only one answer is correct at a time.** If you do not know which answer is correct, please indicate "do not know".

1. **Which treatment option is the first choice for melanoma with metastases and no mutation in the BRAF gene?**

❍ Radiotherapy

❍ Immunotherapy

❍ Chemotherapy

❍ I don‘t know

2. **Which statement about the BRAF mutation (genetic change in melanoma) is correct?**

❍ A BRAF mutation leads to pulmonary metastases.

❍ If there is a mutation of the BRAF gene, the immune therapies are not effective.

❍ If there is a mutation of the BRAF gene, therapy with pills is possible.

❍ I don‘t know

3. **During immunotherapy with the so-called checkpoint blockers, patients receive infusion therapy. How does this work?**

❍ The infusion kills individual tumor cells in the blood. This prevents tumor cells from entering into other tissues and developing metastases there.

❍ The substances in the infusion stimulate the body's immune system. Thus the tumor is detected and fought more effectively.

❍ The infusion contains toxic substances that disrupt the metabolism of the tumour cells. This prevents further division of the tumor cells.

❍ I don‘t know

Study code + date (*filled in by staff*)

In the following seven questions on immunotherapy, you should tick whether the answer options are correct or incorrect. **There is always at least one correct answer. However, several or all statements can also be correct.** If you do not know whether an answer is right or wrong, please indicate "do not know".

Place a cross behind each answer option. For example:

Welche Aussage(n) zu den Vorfahrtsregeln ist/sind richtig?

	richtig	*falsch*	*weiß nicht*
Wenn keine Ampeln oder Verkehrszeichen angebracht sind, gilt die Regel rechts vor links.	X	❍	❍
Sind gleichzeitig Ampeln und Verkehrszeichen angebracht, muss man sich nach den Verkehrszeichen richten.	❍	X	❍
Die Rechts-Vor-Links-Regel gilt nicht bei Ausfahrten eines Grundstücks.	❍	❍	X
An einem Stopp-Schild muss man anhalten und Vorfahrt gewähren.	X	❍	❍

4. Which statement(s) on the course of immunotherapy is/are correct?

	right	*wrong*	*don't know*
The active substances of the immunotherapy are administered by infusion, which is done every 2 days.	❍	❍	❍
The active substances of the immunotherapy are administered by infusion, which takes place every 2-6 weeks.	❍	❍	❍
To receive the infusion, patients must be admitted as inpatients and stay in hospital for one night for monitoring.	❍	❍	❍
During immunotherapy, staging examinations (imaging by CT/MRT) are performed approximately every 3 months.	❍	❍	❍

Study code + date (*filled in by staff*)

5. Which statement(s) on mono-immunotherapy is/are correct?

	right	*wrong*	*don't know*
Monotherapy leads to tumor control (i.e. the same or reduced tumor size) in slightly **less** than 50% of patients.	❍	❍	❍
Monotherapy leads to tumor control (i.e. the same or reduced tumor size) in slightly **over** 60% of patients.	❍	❍	❍
**More** than half of the patients with monotherapy must be treated for severe side effects.	❍	❍	❍
**Less** than a quarter of patients with monotherapy must be treated for severe side effects.	❍	❍	❍

6. Which statement(s) on combined immunotherapy is/are correct?

	right	*wrong*	*don't know*
Combination therapy leads to tumor control (i.e. the same or reduced tumor size) in slightly **less** than 50% of patients.	❍	❍	❍
Combination therapy leads to tumor control (i.e. the same or reduced tumor size) in slightly **over** 60% of patients.	❍	❍	❍
**More** than half of the patients with combination therapy must be treated for severe side effects.	❍	❍	❍
**Less** than a quarter of patients with combination therapy must be treated for severe side effects.	❍	❍	❍

7. Which statement(s) on benefits and risks of immunotherapy is/are correct?

	right	*wrong*	*don't know*
Under combination therapy, the proportion of patients in whom the tumor becomes smaller is higher than under monotherapy.	❍	❍	❍
Under combination therapy, the proportion of patients in whom the disease continues to progress in the longer term is slightly higher than under monotherapy.	❍	❍	❍
Mild side effects occur with approximately the same frequency in mono- and combination therapy.	❍	❍	❍
Severe side effects occur more frequently with combination therapy than with monotherapy.	❍	❍	❍

8. What mild side effect(s) is/are typical for immunotherapy?

	right	*wrong*	*don't know*
Skin rash	❍	❍	❍
Fatigue	❍	❍	❍
Hair loss	❍	❍	❍
Joint pain	❍	❍	❍

9. Which severe side effect(s) is/are typical for immunotherapy?

	right	*wrong*	*don't know*
Psychoses	❍	❍	❍
Intestinal inflammation or diarrhoea	❍	❍	❍
Inflammation of thyroid gland	❍	❍	❍
Inflammation of the liver	❍	❍	❍

UniversitätsKlinikum Heidelberg

NATIONALES CENTRUM FÜR TUMORERKRANKUNGEN HEIDELBERG

getragen von:
Deutsches Krebsforschungszentrum
Universitätsklinikum Heidelberg
Thoraxklinik-Heidelberg
Deutsche Krebshilfe

Study code + date (*filled in by staff*)

10. Which statement(s) about the side effects of immunotherapy is/are correct?

	right	*wrong*	*don't know*
Regular blood checks are recommended to detect side effects.	❍	❍	❍
If immunotherapy has to be discontinued because of side effects, its effectiveness is not reduced.	❍	❍	❍
If the immunotherapy is discontinued, the side effects always disappear.	❍	❍	❍
Mild side effects can be treated symptomatically (e.g. skin side effects with an ointment) and the immunotherapy can be continued.	❍	❍	❍

Finally, we are interested in how well informed you feel about your treatment options. To do this, place a cross on the scale.

Example: How concerned are you about climate change?

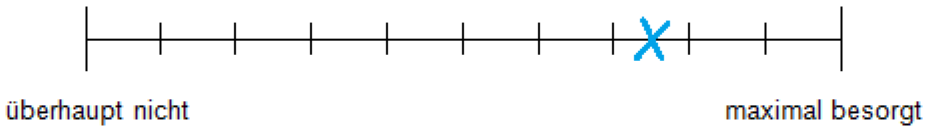

11. How well informed do you currently feel about your treatment options?

Not at all — maximally informed
