# Supplementary material for: Development and validation of a web-based patient decision aid for immunotherapy for patients with metastatic melanoma: study protocol for a multicenter randomized trial
Source: Trials. 2021 Apr 20;22:294. doi: 10.1186/s13063-021-05234-4 (PMC8056554; doi:10.1186/s13063-021-05234-4)
Supplement: Supplementary file 4 — Additional file 4. Copy of the original ethical approval document including an English translation. [file 13063_2021_5234_MOESM4_ESM.pdf]

MEDIZINISCHE
FAKULTÄT
**HEIDELBERG**

Ethikkommission der Med. Fak. HD | Alte Glockengießerei 11/1 | 69115 Heidelberg

Frau Prof. Dr. med. Christiane Bieber
Universitätsklinikum Heidelberg
Klinik für Allgemeine Innere Medizin
und Psychosomatik
Thibautstr. 4
69115 Heidelberg

**Universität Heidelberg**
**Ethikkommission der**
**Med. Fakultät**

Alte Glockengießerei 11/1
69115 Heidelberg

Tel. +49 6221 56264-60 (Zentrale)
Fax +49 6221 56264-80
ethikkommission-I@med.uni-heidelberg.de

www.medizinische-fakultaet-hd.uni-heidelberg.de/ethikkommission

**Vorsitz:**
Prof. Dr. med. Dr. h.c. Thomas Strowitzki

**Stellv. Vorsitz:**
Prof. Dr. med. Johannes Schröder
Prof. Dr. med. Klaus Herfarth

**Geschäftsleitung:**
Dr. med. Verena Pfeilschifter

eFeedback
Scan or click

04.07.2019
ts-al/si

**BERUFSRECHTLICHE BERATUNG**

*Unser Zeichen:* **S-436/2019 (Bitte stets angeben)**
*Titel:* **PEF-Immun: „Partizipative Entscheidungsfindung zur Immuntherapie in der Onkologie – prospektive, randomisiert kontrollierte Studie“**

Sehr geehrte Frau Professor Bieber,

die Ethikkommission hat Ihr Forschungsvorhaben in der Sitzung am 01.07.2019 beraten und hat keine Bedenken gegen die Durchführung der Studie.

Sie gibt jedoch folgende Empfehlungen bzw. Hinweise:

**Allgemein:**

1. In der Einwilligungserklärung (S. 1) und im Studienprotokoll (S. 17) wird erwähnt, dass pseudonymisierte Studiendaten ggf. weitergegeben werden. Laut Patienteninformation erfolgt jedoch keine Datenweitergabe. Die Angaben sind in den Dokumenten zu vereinheitlichen. Sofern eine Weitergabe von pseudonymisierten Daten erfolgt, sind die Empfänger so genau wie möglich zu nennen. Ist eine konkrete Angabe nicht möglich, so sind zumindest die möglichen Kategorien von Empfängern (z.B. Universitäten, Kliniken etc.) zu nennen (vgl. Art. 13 Abs. 1e DSGVO).
2. Im Studienprotokoll auf Seite 13 wird beschrieben, dass neben den Mitarbeitern der Studie auch Mitarbeiter des Kooperationspartners TAKEPART Media + Science GmbH Zugriff auf die Studiendaten haben. Die Studienteilnehmer sind darüber in der Informationsschrift zu informieren und müssen diesem Vorgehen in der Einwilligungserklärung zustimmen.
3. Die studienspezifischen Fragebögen bzw. Entscheidungshilfen, die im Rahmen der Studie entwickelt werden sollen (MC-Wissenstest, Subjektive Risikobewertung, PtDA), sind der Ethikkommission der Medizinischen Fakultät Heidelberg nachzureichen, sobald diese vorliegen.

**Studienprotokoll:**

4. Unklare Fragestellung und fehlende statistische Hypothese. „Für die genannten Hypothesen, die mittels Regressionsanalysen untersucht werden, ist diese angestrebte Stichprobengröße nach Field (2013) ausreichend.“ Dies ist eine unzureichende Begründung der Fallzahl und die Referenz fehlt.

Qualitätsmanagement DQS ISO 9001:2015 399893 QM 15

BIC SOLADEST600
IBAN DE64 6005 0101 7421 5004 29

MF HD

5. S. 17, Abschnitt 11: Neben dem Bundesdatenschutzgesetz sind auch die Bestimmungen der Datenschutz-Grundverordnung (DSGVO) sowie des Landesdatenschutzgesetz Baden-Württemberg (LDSG BW) einzuhalten.

**Informationsschrift für Patientinnen und Patienten:**

6. S. 2 f., Abschnitt „b) Kontrollgruppe", siehe Satz *„Als Aufwandsentschädigung erhalten die Patienten der Kontrollgruppe einen Verzehrgutschein der Cafeteria des NCT Heidelberg."*: Weshalb erhalten nur Patienten der Kontrollgruppe einen Verzehrgutschein der Cafeteria? Dieser sollte an alle Studienteilnehmer ausgehändigt werden. Zudem sollte angegeben werden, in welchen Wert der Gutschein hat.

Wir wünschen Ihnen bei der Durchführung der Studie viel Erfolg.

Bitte leiten Sie das Ergebnis der berufsrechtlichen Beratung und die studienrelevante Korrespondenz allen teilnehmenden Ärzten in unserem Zuständigkeitsbereich weiter.

Mit freundlichen Grüßen

i.V. Digital unterschrieben von Dr. Pfeilschifter, Verena Datum: 2019.07.04 14:23:47 +02'00'

Prof. Dr. med. Dr. h.c. Thomas Strowitzki
Vorsitzender

**Anlagen**
Anhang

**Allgemeine Hinweise:**

- Änderungen in Organisation und Ablauf der Studie sind der Kommission, zusammen mit einer Bewertung der Nutzen-Risiko-Relation, umgehend mitzuteilen. Sowohl die **Antragsnummer** als auch die **geänderten Passagen** sollten in den betreffenden Unterlagen **deutlich gekennzeichnet** sein, da anderenfalls keine zügige Bearbeitung möglich ist.
- Innerhalb von einem Jahr nach Studienende sollte die Studienleitung der Kommission einen Abschlussbericht vorlegen, der eine Zusammenfassung der Ergebnisse und Schlussfolgerungen der Studie enthält, unabhängig davon, ob diese vollständig abgeschlossen oder vorzeitig beendet wurde. Dafür ist die auf der Homepage der Kommission abrufbare Mustervorlage „Abschlussbericht" zu verwenden (Pfad: -> Sonstige Studien -> Vorlagen).
- Jedes Forschungsvorhaben, an dem Versuchspersonen beteiligt sind, ist vor der Rekrutierung der ersten Versuchsperson in einer öffentlich zugänglichen Datenbank zu registrieren.
- Die Ethikkommission der Medizinischen Fakultät Heidelberg arbeitet gemäß den nationalen gesetzlichen Bestimmungen und den ICH-GCP-Richtlinien. Ihren Beratungen liegt die Deklaration des Weltärztebundes von Helsinki in der jeweils aktuellen Fassung zugrunde.
- Unabhängig vom Beratungsergebnis macht die Ethikkommission Sie darauf aufmerksam, dass die ethische und rechtliche Verantwortung für die Durchführung einer Studie beim Leiter der Studie und bei allen teilnehmenden Ärzten liegt.
- Datenschutzrechtliche Aspekte von Forschungsvorhaben werden durch die Ethikkommission grundsätzlich nur kursorisch geprüft. Dieses Votum / diese Bewertung ersetzt mithin nicht die Konsultation des zuständigen Datenschutzbeauftragten. Die Einhaltung der einschlägigen Datenschutzgesetze sowie die Umsetzung des Datenschutzkonzeptes liegen in der Verantwortung des Studienleiters/Prüfers bzw. Sponsors.



©EKHD-ZB-S-DV-070-190501

**Anhang**

**Liste der eingereichten Unterlagen**

| | |
|---|---|
| **Primär eingereichte Unterlagen:** | Anschreiben vom 31.05.2019<br>Zusammenfassung<br>Checkliste Sonstige Studien<br>Formular für Erstantrag<br>Informationsschrift für Patientinnen und Patienten Version 1<br>Einwilligungserklärung Version 1<br>Studienprotokoll Version 1 vom 31.05.2019<br>Anhang 1: Aufbau der PtDA zur Immuntherapie<br>CV Prof. (apl.) Dr. med. Christiane Bieber (ohne Unterschrift / Datum)<br>TAKEPART Media + Science GmbH |
| **Formelle Nachreichung:** | Nachreichung vom 04.07.2019<br>CV Prof. (apl.) Dr. med. Christiane Bieber vom 04.07.2019 |



©EKHD-ZB-S-DV-070-190501

MEDIZINISCHE
FAKULTÄT
HEIDELBERG

Ethikkommission der Med. Fak. HD Alte Glockengießerei 11/1 69115 Heidelberg

Frau Prof. Dr. med. Christiane Bieber
Universitätsklinikum Heidelberg
Klinik für Allgemeine Innere Medizin
und Psychosomatik
Thibautstr. 4
69115 Heidelberg

**Universität Heidelberg**
**Ethikkommission der**
**Med. Fakultät**

Alte Glockengießerei
11/1 69115 Heidelberg

Tel. +49 6221 56264-60 (Zentrale) Fax
+49 6221 56264-80 ethikkommission-I@med.uni-heidelberg.de

www.medizinische-fakultaet-hd.uni-heidelberg.de/ethikkommission

**Presidency:**
Prof. Dr. med. Dr. h.c. Thomas Strowitzki

**Co Presidency:**
Prof. Dr. med. Johannes Schröder
Prof. Dr. med. Klaus Herfarth

**Management:**
Dr. med. Verena Pfeilschifter

eFeedback
Scan or click

04.07.2019
ts-al/si

**PROFESSIONAL LEGAL ADVICE**

***Our sign:*** **S-436/2019 (Please always indicate)**

***Titel:*** **PEF-Immun:**
**"Participatory decision making on immunotherapy in oncology - prospective, randomized controlled trial"**

Dear Professor Bieber,

the ethics committee has discussed your research project in the meeting on 01.07.2019 and has no objections to the conduct of the study

However, it makes the following recommendations and advice:

**In general:**

1. The declaration of consent (p. 1) and the study protocol (p. 17) mention that pseudonymised study data may be passed on. According to the patient information, however, no data are passed on. The information must be standardised in the documents. If pseudonymised data are passed on, the recipients should be named as precisely as possible. If it is not possible to give specific details, at least the possible categories of recipients (e.g. universities, hospitals, etc.) must be named (cf. Art. 13 Para. 1e DSGVO).
2. The study protocol on page 13 describes that, in addition to the study staff, employees of the cooperation partner TAKEPART Media + Science GmbH also have access to the study data. The study participants must be informed about this in the information leaflet and must agree to this procedure in the declaration of consent.
3. The study-specific questionnaires or decision-making aids to be developed within the framework of the study (MC knowledge test, subjective risk assessment, PtDA) are to be submitted to the Ethics Committee of the Medical Faculty of Heidelberg as soon as they are available.

**Study protocol:**

4. Unclear questions and lack of statistical hypothesis. "For the above hypotheses, which are investigated by means of regression analyses, this targeted sample size according to Field (2013) is sufficient." This is an insufficient justification of the number of cases and the reference is missing.

5. p. 17, section 11: In addition to the Federal Data Protection Act, the provisions of the Basic Data Protection Regulation (DSGVO) and the Baden-Württemberg State Data Protection Act (LDSG BW) must also be observed.

**Information leaflet for patients:**

6. p. 2 f., section "b) Control group", see sentence "As compensation for expenses, the patients of the control group receive a meal voucher from the cafeteria of the NCT Heidelberg": Why do only patients in the control group receive a meal voucher from the cafeteria? This voucher should be given to all study participants. In addition, the value of the voucher should be indicated.

We wish you success in conducting the study.

Please forward the results of the professional consultation and the study-related correspondence to all participating physicians in our area of responsibility.

With kind regards

i.V.

Digital unterschrieben von Dr. Pfeilschifter, Verena
Datum: 2019.07.04
14:23:47 +02'00'

Prof. Dr. med. Dr. h.c. Thomas Strowitzki
Chairman

**Attachments**
Appendix

**General notes:**

- Any changes in the organisation and conduct of the study, together with an assessment of the risk-benefit balance, shall be immediately notified to the Commission. Both the application number and the amended passages should be clearly marked in the relevant documents, otherwise it will not be possible to process them expeditiously.
- Within one year of the end of the study, the study director should submit a final report to the Commission, summarising the results and conclusions of the study, whether fully completed or prematurely terminated. For this purpose, the sample template "Final Report" available on the Commission's website should be used (path: -> Other studies -> Templates).
- Any research project involving subjects shall be registered in a publicly accessible database before the recruitment of the first subject.
- The Ethics Committee of the Medical Faculty of Heidelberg works according to national legal regulations and the ICH-GCP guidelines. Its deliberations are based on the Declaration of the World Medical Association of Helsinki in its current version.
- Regardless of the outcome of the consultation, the Ethics Committee draws your attention to the fact that the ethical and legal responsibility for conducting a study lies with the study director and all participating physicians.
- Data protection aspects of research projects are generally only examined cursory by the ethics committee. This vote/evaluation does not therefore replace consultation with the responsible data protection officer. Compliance with the relevant data protection laws and the implementation of the data protection concept are the responsibility of the study director/examiner or sponsor.



©EKHD-ZB-S-DV-070-190501

## Attachments

### List of documents submitted

| | |
|---|---|
| **Primary submitted Documents:** | Letter of 31.05.2019<br>Summary<br>Checklist other studies<br>Form for initial application<br>Information leaflet for patients Version 1<br>Declaration of Consent Version 1<br>Study protocol version 1 of 31.05.2019<br>Annex 1: Structure of the PtDA for immunotherapy<br>CV Prof. (apl.) Dr. med. Christiane Bieber (without signature / date)<br>TAKEPART Media + Science GmbH |
| **Formal Submission:** | Subsequent submission from 04.07.2019<br>CV Prof. (apl.) Dr. med. Christiane Bieber from 04.07.2019 |



©EKHD-ZB-S-DV-070-190501

Ethikkommission an der TU Dresden
Fetscherstraße 74, 01307 Dresden

Frau
Prof. Friedegund Meier
Klinik und Poliklinik für Dermatologie
Universitätsklinikum Carl Gustav Carus Dresden
**-Hauspost-**

*nachrichtlich: christiane.bieber@med.uni-heidelberg.de*

PD Dr. med.
Hermann Theilen
Vorsitzender der Ethikkommission

Telefon: 0351 458-2992
Telefax: 0351 458-4369
E-Mail: ethikkommission@mailbox.tu-dresden.de

Dresden, 12.03.2020

## Auflagenerfüllung

**Studie:** **Partizipative Entscheidungsfindung zur Immuntherapie in der Onkologie – prospektive, randomisiert kontrollierte Studie (PEF-Immun)**

**Unser AZ:** **EK 529122019** *(bitte stets angeben!)*

**Antragsteller:** **Klinik und Poliklinik für Dermatologie, Prof. Meier**

Sehr geehrte Frau Professor Meier,

mit den ergänzenden Unterlagen zu o. g. Studie, die am 06. und 09.03.2020 hier eingegangen sind, wurden die Auflagen aus unserem Schreiben vom 06.02.2020 erfüllt.

Nach Auffassung der Ethikkommission an der Technischen Universität Dresden bestehen nunmehr gegen das Forschungsvorhaben

☒ keine Bedenken.

☐ keine Bedenken. Die im Einzelnen aufgeführten weiteren Hinweise bzw. Empfehlungen[1] sollten jedoch berücksichtigt werden. Eine erneute Vorlage der überarbeiteten Unterlagen ist nicht notwendig.

[1] *Hinweise und Empfehlungen sollen auf die moralische und juristische Verpflichtung hinweisen und in freier Entscheidung und Verantwortung zur Überprüfung und Anpassung der Studiendokumente anregen.*

Die allgemeinen Hinweise aus der mit Auflagen versehenen Erstberatung / Erstbewertung gelten entsprechend und sind zu berücksichtigen.

Die Ethikkommission an der TU Dresden stützt sich bei der Beurteilung der eingereichten Studienunterlagen insbesondere auf die Richtlinien der Deklaration des Weltärztebundes von Helsinki in der jeweils geltenden Fassung, auf die strahlenschutzrechtlichen Vorschriften und auf die allgemein anerkannten Richtlinien für „Good Clinical Practice" (GCP).

Die Arbeitsweise und die Zusammensetzung der Ethikkommission entspricht den jeweils geltenden gesetzlichen Regelungen bzw. Empfehlungen.

Es wird bestätigt, dass keine Mitglieder der Ethikkommission, die am o. g. Forschungsvorhaben beteiligt sind, am Beratungs- bzw. Bewertungsergebnis mitgewirkt haben oder in anderer Form beteiligt gewesen sind.

Wir wünschen Ihnen bei der Durchführung Ihres Forschungsvorhabens viel Erfolg.

Mit freundlichen Grüßen

Ass. jur. Julia Steinigen
i. A. Juristin [illegible]
geschäftsführendes Mitglied
der Ethikkommission
an der Technischen Universität Dresden
Fetscherstraße 74
01307 Dresden

PD Dr. med. H. Theilen
Vorsitzender der Ethikkommission

TECHNISCHE
UNIVERSITÄT
DRESDEN

Ethikkommission

Ethikkommission an der TU Dresden
Fetscherstra ße 74, 01307 Dresden

PD Dr. med.
Hermann Theilen
Chairman of the Ethics Committee

Telefon: 0351 458-2992
Telefax: 0351 458-4369
E-Ma il: ethikkommission@mailbox.tu-dresden.de

Mrs.
Prof. Friedegund Meier
Clinic and Polyclinic for Dermatology
Universitätsklinikum Carl Gustav Carus Dresden
-house mail-

*by message: christiane. bieber4.med uni-heldelberz de*

Dresden, 12.03.2020

# Compliance

Study: Participatory decision-making on immunotherapy in oncology - prospective randomized controlled trial (PEF-Immune)

Our AZ: EK 529122019 *(please always indicate!)*

Applicant: Clinic and Polyclinic for Dermatology, Prof. Meier

Dear Professor Meier,

with the supplementary documents to the above-mentioned study, which were received here on 06. and 09.03.2020, the requirements of our letter of 06.02.2020 were fulfilled.

In the opinion of the ethics commission at the Technical University of Dresden, the research project is now opposed by

[X] no qualms.

[ ] no qualms. However, the further advice or recommendations* listed in detail should be considered. It is not necessary to resubmit the revised dossier.

---

*References and recommendations should point out the moral and legal obligation and should be made in free decision and Encourage responsibility for reviewing and adapting the study documents.*

The general instructions from the initial consultation / initial assessment with conditions apply accordingly and must be taken into account.

The Ethics Committee at the TU Dresden bases its assessment of the submitted study documents in particular on the guidelines of the Declaration of the World Medical Association of Helsinki in the currently valid version, on the radiation protection regulations and on the generally accepted guidelines for
"Good Clinical Practice (GCP).

The working methods and composition of the Ethics Committee comply with the applicable legal regulations and recommendations.

It is confirmed that no members of the Ethics Committee who are involved in the above-mentioned research project have contributed to the results of the consultation or evaluation or have been involved in any other way.

We wish you every success in your research project.

With kind regards

Ass. jur. Julia Steinigen
i.A. [illegible]-Fuchs
Juristin
geschäftsführendes Mitglied
der Ethikkommission
an der Technischen Universität Dresden
Fetscherstraße 7[illegible]
01307 Dresden

PD Dr. med. H. Theilen
Chairman of the Ethics Committee
