# Supplementary material for: Development and validation of a web-based patient decision aid for immunotherapy for patients with metastatic melanoma: study protocol for a multicenter randomized trial
Source: Trials. 2021 Apr 20;22:294. doi: 10.1186/s13063-021-05234-4 (PMC8056554; doi:10.1186/s13063-021-05234-4)
Supplement: Supplementary file 5 — Additional file 5. Copy of the original funding document including an English translation. [file 13063_2021_5234_MOESM5_ESM.pdf]

Gemeinsamer Bundesausschuss
Innovationsausschuss

Innovationsausschuss beim G-BA, Postfach 12 06 06, 10623 Berlin

Universitätsklinikum Heidelberg
Klinik für Allgemeine Innere Medizin und Psychosomatik
Frau Dr. Christiane Bieber
Thibautstraße 4
69115 Heidelberg

Besuchsadresse:
Wegelystr. 8
10623 Berlin

Ansprechpartner/in beim DLR-PT:
Dr. Sabrina Gollos

Telefon:
+49 228 3821-2141

Telefax:
+49 228 3821-1257

E-Mail:
sabrina.gollos@dlr.de

Datum:
23. November 2018

Förderbescheid

Förderung aus Mitteln des Innovationsfonds zur Förderung von Versorgungsforschung (§ 92a Abs. 2 Satz 1 SGB V) für das Projekt:
„PEF-Immun – Partizipative Entscheidungsfindung zur Immuntherapie in der Onkologie – prospektive, randomisiert kontrollierte Studie“
Förderkennzeichen: 01VSF18047

Projektleitung: Dr. Christiane Bieber

Ihr Antrag mit Eingang am 19. Februar 2018
mit Ergänzungen vom 23. August 2018, 17. Oktober 2018 (Schreiben und E-Mail), 5. November 2018 (Schreiben und E-Mail) und 8. November 2018 (E-Mail)

Sehr geehrte Frau Dr. Bieber,

mit dem vorliegenden Förderbescheid wird aufgrund der Entscheidung des Innovationsausschusses vom 16. August 2018 die folgende Projektförderung aus Mitteln des Innovationsfonds gewährt.

I. Genehmigung der Europäischen Kommission

Gemäß Nr. 2.1 des Unionsrahmens für staatliche Beihilfen zur Förderung von Forschung und Entwicklung und Innovation („FuEuI-Rahmen“ - Mitteilung der Kommission vom 27. Juni 2014, ABl. EU C 198/1) sind Einrichtungen für Forschung und Wissensverbreitung („Forschungseinrichtungen“) und Forschungsinfrastrukturen Empfänger staatlicher Beihilfen i. S. v. Artikel 107 Absatz 1 AEUV, wenn sie unternehmerisch handeln. Der Unternehmenscharakter hängt nicht von der Rechtsform (öffentlich-rechtlich oder privatrechtlich) oder dem wirtschaftlichen Charakter (gewinnorientiert oder nicht) ab. Entscheidend ist vielmehr, ob die Forschungseinrichtung eine wirtschaftliche Tätigkeit ausübt, d. h. ob sie auf einem bestimmten Markt Produkte oder Dienstleistungen anbietet. Nichtwirtschaftliche Tätigkeiten sind im Allgemeinen primäre Tätigkeiten von Forschungseinrichtungen und Forschungsinfrastrukturen (z. B. die Ausbildung von mehr oder besser qualifizierten Humanressourcen; unabhängige Forschung und Entwicklung zur Erweiterung des Wissens und des Verständnisses; weite Verbreitung der Forschungsergebnisse auf nichtausschließlicher und nichtdiskriminierender Basis; Tätigkeiten des Wissenstransfers). Übt ein und dieselbe Forschungseinrichtung sowohl wirtschaftliche als auch nichtwirtschaftliche Tä-

tigkeiten aus, fällt die öffentliche Finanzierung der nichtwirtschaftlichen Tätigkeiten nicht unter Artikel 107 Abs. 1 AEUV, wenn die nichtwirtschaftlichen Tätigkeiten und ihre Kosten, Finanzierung und Erlöse klar voneinander getrennt werden können, sodass keine Gefahr der Quersubventionierung der wirtschaftlichen Tätigkeiten besteht. Es ist deshalb im Einzelfall festzustellen, ob eine Forschungseinrichtung eine wirtschaftliche oder eine nichtwirtschaftliche Tätigkeit ausübt:

Das Universitätsklinikum Heidelberg und die Technische Universität Dresden handeln im nichtwirtschaftlichen Bereich (Ziffer 2.1 Nr. 19 des Unionsrahmens). Die Förderung dieser Einrichtungen bedurfte keiner Genehmigung durch die Europäische Kommission.

II. Förderzweck, Umfang der Förderung und Zahlungsplan

Aufgrund Ihres Antrags mit Eingang am 19. Februar 2018 mit o. a. Ergänzungen wird Ihnen gemäß § 92a Abs. 2 SGB V sowie der Verfahrensordnung des Innovationsausschusses beim Gemeinsamen Bundesausschuss für die Zeit

vom 1. Januar 2019 bis 31. Dezember 2021 (Förderzeitraum)

im Rahmen einer **Vollfinanzierung** ein nicht rückzahlbarer Förderbetrag von

bis zu **574.677,44 €**
(in Buchstaben: Fünf-sieben-vier-sechs-sieben-sieben-Komma-vier-vier Euro),

höchstens jedoch in Höhe der förderfähigen Ausgaben bewilligt.

Die Fördermittel sind zweckgebunden und dürfen nur für die im Förderzeitraum verursachten Ausgaben für das o. a. Projekt abgerechnet werden.

Die Bewilligung setzt voraus, dass die Gesamtfinanzierung des Projekts gesichert bleibt.

Der als Anlage F beigefügte Finanzierungsplan einschließlich des Teilfinanzierungsplans ist als Bestandteil des Förderbescheids verbindlich.

Bitte beachten Sie, dass aus dem Teilfinanzierungsplan des Konsortialpartners Technische Universität Dresden Mittel aus der Position Sonstige Sachausgaben in Höhe von 962,00 € in die Position Aufträge an Dritte der Konsortialführung verschoben wurden. Des Weiteren wurden Mittel von 242,00 € aus der Position Sonstige Sachausgaben des Finanzierungsplans der Konsortialführung in die Position Aufträge an Dritte des Finanzierungsplans der Konsortialführung verschoben.

Der Differenzbetrag von 21,00 € zwischen der maximalen Fördersumme und der ausgewiesenen Gesamtsumme im überarbeiteten Finanzierungsplan wurde in die Position Sachausgaben (Sonstige Sachausgaben) der Konsortialführung eingestellt.

Der Anpassung der Fallzahl wird zugestimmt.

III. Nebenbestimmungen

Bestandteil dieses Förderbescheids sind die beigefügten Allgemeinen Nebenbestimmungen des Innovationsausschusses beim Gemeinsamen Bundesausschuss für Förderungen aus dem Innovationsfonds (ANBest-IF, Anlage N) sowie die nachstehenden besonderen Nebenbestimmungen:

1. Meilensteinplan

Der Förderempfänger hat mit dem ersten Statusbericht einen aktualisierten Meilensteinplan für das Projekt entsprechend dem beigefügten Vordruck (Anlage M) vorzulegen, der um die Meilensteine „positives Ethikvotum für den Standort Heidelberg liegt vor“ sowie „positives Ethikvotum für den Standort Dresden liegt vor“ erweitert wurde.
Im Meilensteinplan muss in der Regel mindestens ein relevanter Meilenstein in jedem Quartal der Projektlaufzeit vorhanden sein. Mit der Vorlage des Meilensteinplans ist zudem ein aktuelles Gantt-Chart zur Arbeits- und Zeitplanung vorzulegen. Der Meilensteinplan, das Gantt-Chart und der Zeitplan zur Fallzahlerreichung müssen widerspruchsfrei übereinstimmen. Die Darstellung der Quartale im Gantt-Chart muss sich auf die Jahre der Projektlaufzeit beziehen, damit der Projektfortschritt und die Meilensteinerreichung nachvollziehbar sind.

2. Weiterleitung von Fördermitteln

Der Förderempfänger wird ermächtigt, Teile der Fördermittel an den im Finanzierungsplan (Anlage F) genannten Konsortialpartner weiterzuleiten. Die Regelungen der Nr. 1 ANBest-IF sowie die Anlage W sind zu beachten. Die in diesem Förderbescheid mit * markierten Nebenbestimmungen sind für alle betreffenden Konsortialpartner verbindlich.

3. E-Health Lösungen/Telemedizin*

Nach § 291d SGB V sind bei der Nutzung von informationstechnischen Systemen die relevanten Festlegungen zu beachten und insbesondere die offenen Schnittstellen und die Interoperabilität zu gewährleisten. Bei der Verwendung von elektronischen Anwendungen sind die Regelungen zum Interoperabilitätsverzeichnis nach § 291e Abs. 10 SGB V zu berücksichtigen.

Bitte beachten Sie, dass die Gesellschaft für Telematik die sicheren Verfahren zur Übermittlung medizinischer Dokumente über die Telematikinfrastruktur nach § 291b Absatz 1e SGB V verbindlich festgelegt hat (https://fachportal.gematik.de/spezifikationen/sichere-uebermittlungsverfahren/). Durch den Förderempfänger ist zu prüfen, ob das Projekt betroffen ist und ggf. Anpassungen vorzunehmen sind.

Bei der Nutzung weiterer über die Anwendungen der elektronischen Gesundheitskarte (eGK) hinausgehender elektronischer Anwendungen des Gesundheitswesens sowie der Gesundheitsforschung müssen zudem die Anforderungen nach § 291a Abs. 7 Satz 3 SGB V sowie die Nutzungsvoraussetzungen der gematik nach § 291b Abs. 1b Satz 3 SGB V erfüllt werden (siehe gematik-Homepage
https://fachportal.gematik.de/fileadmin/user_upload/fachportal/files/Spezifikationen/Weitere-Anwendungen/gemRL_NvTIwA_V1.3.0.pdf). Das Bestätigungsverfahren bei der gematik gemäß § 291b Abs. 1b Satz 4 SGB V muss spätestens zum Projektstart abgeschlossen sein.

4. Datenschutz*

Die rechtlichen Rahmenbedingungen für die Verarbeitung personenbezogener Patientendaten, die sich insbesondere aus den Vorschriften der Datenschutz-Grundverordnung, des Bundesdatenschutzgesetzes (BDSG), des SGB V und SGB X ergeben, sind zu beachten. In Zweifelsfällen sind die für den Datenschutz zuständigen Stellen einzuschalten.

5. Ethische Richtlinien*

Bei der Durchführung von Untersuchungen am Menschen und/oder der Gewinnung bzw. Verwendung von menschlichem Probenmaterial im Rahmen dieses Projekts sind die Empfehlungen der Deklaration von Helsinki sowie die Richtlinien des CIOMS (Council for International Organization of Medical Sciences) und der WHO (World Health Organization): „Proposed International Guidelines For Biomedical Research Involving Human Subjects“ in den jeweils geltenden Fassungen einzuhalten.

6. Ethikvotum*

Vor Beginn der Untersuchungen am Menschen und/oder der Gewinnung bzw. Verwendung von menschlichem Probenmaterial ist das uneingeschränkt positive Votum der zuständigen Ethikkommission vorzulegen. Falls diese ein Votum nicht für erforderlich hält, ist eine entsprechende Erklärung der Ethikkommission vorzulegen.

7. Widerrufsvorbehalt*

Dieser Bescheid kann in einem der folgenden Fälle widerrufen und die Förderung ganz oder teilweise eingestellt werden (Widerrufsvorbehalt nach § 32 Abs. 2 Nr. 3 in Verbindung mit § 47 Abs. 1 Nr. 1 SGB X):

- im Fall, dass der Förderzweck nicht zu erreichen ist,
- in den Fällen einer Mittelsperre für Einzelansätze des Finanzierungsplans,
- in den Fällen einer nicht fristgerechten bzw. unvollständigen Vorlage von Nachweisen,
- aus zwingenden Gründen.

8. Nachweis der Verwendung*

Für den Nachweis der Verwendung gelten die Regelungen der Nr. 14 ANBest-IF. Die entsprechenden Vordrucke werden dem Förderempfänger zu gegebener Zeit zur Verfügung gestellt. Für die Belegliste ist der Vordruck nach Anlage B zu verwenden.

9. Projektveranstaltungen

Der Förderer ist über wichtige Termine bzw. Veranstaltungen, Newsletter usw. zu informieren bzw. einzuladen. Auf Verlangen ist jederzeit Auskunft über Art, Umfang und Erfolg der durchgeführten Maßnahmen zu geben.

10. Auszahlung der Fördermittel

Die Fördermittel werden auf Anforderung des Förderempfängers entsprechend der Nr. 7 ANBest-IF ausgezahlt. Projektbezogene förderfähige Ausgaben, die nach Förderbeginn entstanden sind, können später mit der Zahlungsanforderung zur Abrechnung eingereicht werden.

Eine Auszahlung von Fördermitteln kann erst erfolgen, wenn der Förderbescheid nach Ablauf der Rechtsbehelfsfrist bestandskräftig geworden ist und die Eingangsbestätigung (Vordruck nach Anlage E) vorgelegt wurde. Der Förderempfänger kann die Bestandskraft des Bescheids vorher herbeiführen, wenn er auf den Rechtsbehelf verzichtet (Vordruck nach Anlage E).

Gemäß Nr. 7 Abs. 1 Satz 2 ANBest-IF kann die erste Zahlungsanforderung bis spätestens 31. Januar 2019 vorgelegt werden. Bitte beachten Sie: Die Zahlungsanforderung ist jeweils innerhalb von vier Wochen nach Quartalsbeginn für das laufende Quartal vorzulegen.

Für die Erstellung der Zahlungsanforderung ist der Vordruck nach Anlage Z zu verwenden und Anlage H zu berücksichtigen. Für den mit der Zahlungsanforderung vorzulegenden Statusbericht (siehe Nr. 7 ANBest-IF) ist der Vordruck nach Anlage S zu verwenden.

Zusätzlich ist mit jeder Zahlungsanforderung über den aktuellen Stand der Fallzahlerreichung für das Projekt anhand des beigefügten Vordrucks (Anlage P) zu berichten.

Konsortialpartner verwenden den Vordruck nach Anlage K für die Anforderung von Mitteln bei der Konsortialführung.

11. Rückzahlungen

Rückzahlungen von Fördermitteln sowie ggf. Zinsen sind unter Angabe des Förderkennzeichens 01VSF18047 auf das nachstehende Bankkonto zu überweisen:

Kontoinhaber:	Gemeinsamer Bundesausschuss
Bank:	Deutsche Apotheker- und Ärztebank eG
IBAN:	DE69 3006 0601 0004 2118 20
BIC:	DAAEDEDDXXX

12. Beauftragung eines Projektträgers

Als Projektträger für den Innovationsausschuss beim Gemeinsamen Bundesausschuss ist gegenwärtig der DLR Projektträger beauftragt, die Projektförderung im Rahmen des Innovationsfonds abzuwickeln.

Alle die Durchführung und Abwicklung des Projekts betreffenden Vorgänge sind an den Projektträger zu senden:

DLR Projektträger
– Bereich Gesundheit –
Heinrich-Konen-Str. 1
53227 Bonn

Fachliche Betreuung:
Dr. Sabrina Gollos
Tel.: +49 228 3821-2141
E-Mail: sabrina.gollos@dlr.de

Administrative Betreuung:
Daniela Hofmann
Tel.: +49 228 3821-1748
E-Mail: daniela.hofmann@dlr.de

Eine **Durchschrift** des Bescheids wird an die Projektleitung sowie die administrative Ansprechperson per E-Mail übersandt.

Mit freundlichen Grüßen

Prof. Josef Hecken
Dieser Bescheid wurde elektronisch erstellt und ist auch ohne Originalunterschrift gültig.

**Anlagen:**	- Allgemeine Nebenbestimmungen des Innovationsausschusses beim Gemeinsamen Bundesausschuss für Förderungen aus dem Innovationsfonds (ANBest-IF), Stand August 2018	Anlage N
	- Eingangsbestätigung und Rechtsbehelfsverzicht	Anlage E
	- Finanzierungsplan einschließlich Teilfinanzierungsplan	Anlage F
	- Vordruck Zahlungsanforderung	Anlage Z
	- Hinweise zum Ausfüllen der Zahlungsanforderung	Anlage H
	- Vordruck Statusbericht zur Zahlungsanforderung	Anlage S
	- Vordruck Zeitplan zur Fallzahlerreichung	Anlage P
	- Vordruck Meilensteinplan	Anlage M
	- Vordruck der Belegliste als Anlage zum Verwendungsnachweis	Anlage B
	- Vordruck Weiterleitungsvertrag	Anlage W
	- Vordruck Zahlungsanforderung für Konsortialpartner	Anlage K
	- G-BA-Logo Innovationsausschuss (nur per E-Mail)	Anlage L
	- Hinweise zur Nutzung des G-BA-Logos Innovationsausschuss	Anlage LH

Rechtsbehelfsbelehrung

Gegen diesen Förderbescheid kann binnen eines Monats nach Bekanntgabe beim Landessozialgericht Berlin-Brandenburg, Försterweg 2-6, 14482 Potsdam, schriftlich oder zur Niederschrift des Urkundsbeamten der Geschäftsstelle Klage erhoben werden. Die Klage kann auch nach den Maßgaben der Verordnung über den elektronischen Rechtsverkehr im Land Brandenburg vom 14.06.2006 (GVBl.II/06, [Nr. 33], S. 558), idF vom 19.12.2017 (GVBl.II/17, [Nr. 73]) unter Verwendung einer qualifizierten elektronischen Signatur in elektronischer Form bei der elektronischen Poststelle des Landessozialgerichts Berlin-Brandenburg über die auf der Internetseite www.erv.brandenburg.de bezeichneten Kommunikationswege eingereicht werden.

Gemeinsamer Bundesausschuss
Innovationsausschuss

Innovationsausschuss beim G-BA, Postfach 12 06 06, 10623 Berlin

Universitätsklinikum Heidelberg
Klinik für Allgemeine Innere Medizin und Psychosomatik
Frau Dr. Christiane Bieber
Thibautstraße 4
69115 Heidelberg

Visiting address:
Wegelystr. 8
10623 Berlin

Contact person at DLR-PT:
Dr. Sabrina Gollos

Telefon:
+49 228 3821-2141

Telefax:
+49 228 3821-1257

E-Mail:
sabrina.gollos@dlr.de

Datum:
23. November 2018

Grant decision

Funding from the Innovation Fund for the Promotion of Health Services Research (§ 92a, subsection 2 line 1 SGB V)) for the project:
"PEF-Immun - Shared decision-making on immunotherapy in oncology - prospective, randomized controlled study".
Funding code: 01VSF18047

Project Lead: Dr. Christiane Bieber

Your application received on 19 February 2018
with amendments dated 23 August 2018, 17 October 2018 (letter and e-mail), 5 November 2018 (letter and e-mail) and 8 November 2018 (e-mail)

Dear Dr. Bieber,

with the present grant decision, the following project funding is granted from the Innovation Fund on the basis of the decision of the Innovation Committee of 16 August 2018.

I. Approval by the European Commission

According to point 2.1 of the Union Framework for State Aid for Research and Development and Innovation ('R&D&I Framework' - Commission Communication of 27 June 2014, OJ EU C 198/1), research and knowledge dissemination organisations ('research organisations') and research infrastructures are recipients of State aid within the meaning of Article 107(1) TFEU if they act in an entrepreneurial manner. The nature of the enterprise does not depend on its legal form (public or private) or its economic character (profit-making or not). Rather, the decisive factor is whether the research institution carries out an economic activity, i.e. whether it offers products or services on a specific market. Non-economic activities are generally primary activities of research organisations and research infrastructures (e.g. training of more or better qualified human resources; independent research and development to increase knowledge and understanding; wide dissemination of research results on a non-

exclusive and non-discriminatory basis; knowledge transfer activities). If one and the same research institution carries out both economic and non-economic activities, public funding of the non-economic activities does not fall under Article 107 (1) TFEU if the non-economic activities and their costs, funding and revenues can be clearly separated so that there is no risk of cross-subsidization of the economic activities. It must therefore be determined on a case-by-case basis whether a research institution is engaged in economic or non-economic activities:

Heidelberg University Hospital and Dresden University of Technology act in the non-economic sphere (point 2.1 no. 19 of the Union framework). The funding of these institutions did not require approval by the European Commission.

II. Purpose of funding, scope of funding and payment schedule

On the basis of your application received on 19 February 2018 with the above-mentioned amendments, you will be informed in accordance with § 92a (2) SGB V and the Rules of Procedure of the Innovation Committee at the Joint Federal Committee for the period

from 1 January 2019 to 31 December 2021 (eligible period)

in the context of **full financing**, a non-repayable grant amount of

up till **574.677,44 €**
(in letters: five-seven-four-six-seven-decimal-four euros),

up to the amount of the eligible expenditure.

The subsidies are earmarked for a specific purpose and may only be used to cover the expenses incurred during the period of funding for the above-mentioned project.

Approval is subject to the condition that the overall financing of the project remains secured.

The financing plan including the partial financing plan attached as Attachment F is binding as an integral part of the funding decision.

Please note that funds from the partial financing plan of the consortium partner Technische Universität Dresden were shifted from the item Other operating expenses in the amount of € 962.00 to the item Orders to third parties of the consortium leadership. In addition, funds of €242.00 were transferred from the item Other operating expenses of the consortium management's financing plan to the item Orders to third parties of the consortium management's financing plan.

The difference of € 21.00 between the maximum funding amount and the total amount shown in the revised financing plan has been entered under the item material expenses (other material expenses) of the consortium management.

The adjustment of the number of cases is approved.

III. Additional provisions

The attached General Ancillary Provisions of the Innovation Committee at the Federal Joint Committee for Funding from the Innovation Fund (ANBest-IF, Annex N) and the following special ancillary provisions form an integral part of this funding decision:

1. Milestone plan

With the first status report, the funding recipient must submit an updated milestone plan for the project in accordance with the attached form (Annex M), which has been expanded to include the milestones "positive ethics vote for the Heidelberg site" and "positive ethics vote for the Dresden site".

As a rule, the milestone plan must contain at least one relevant milestone in each quarter of the project duration. With the submission of the milestone plan, a current Gantt chart for work and time planning must also be submitted. The milestone plan, the Gantt chart and the time schedule for achieving the number of cases must be consistent with each other. The representation of the quarters in the Gantt chart must refer to the years of the project duration, so that the project progress and the milestone achievement are comprehensible.

2. Forwarding of subsidies

The funding recipient is authorised to transfer part of the funding to the consortium partner named in the financing plan (Annex F). The regulations of No. 1 ANBest-IF and Annex W must be observed. The ancillary provisions marked with * in this funding approval are binding for all consortium partners concerned.

3. E-health solutions/telemedicine*

According to § 291d SGB V, the relevant specifications must be observed when using information technology systems and, in particular, open interfaces and interoperability must be ensured. When using electronic applications, the regulations on the interoperability register in accordance with § 291e Para. 10 SGB V must be taken into account.

Please note that the Gesellschaft für Telematik provides the secure procedures for the transmission of medical documents via the telematics infrastructure according to § 291b para. 1e SGB V.
has set out binding rules (https://fachportal.gematik.de/spezifikationen/sichere-uebermittlungsverfahren/). The beneficiary must check whether the project is affected and make any necessary adjustments.

In the use of further applications of the electronic health card (eGK) electronic applications in the health care sector and in the health care sector research must also comply with the requirements of § 291a para. 7 sentence 3 SGB V and the prerequisites for using gematik according to Section 291b (1b) sentence 3 SGB V are met (see homepage of gematik https://fachportal.gematik.de/fileadmin/user_upload/fachportal/files/Spezifikationen/Weitere-Applications/gemRL_NvTIwA_V1.3.0.pdf). The confirmation procedure at gematik according to § Section 291b (1b) sentence 4 SGB V must be completed at the latest at the start of the project.

4. Data protection*

The legal framework conditions for the processing of personal patient data, which result in particular from the regulations of the Data Protection Basic Regulation, the Federal Data Protection Act (BDSG), the Social Code Book V and Social Code Book X, must be observed. In cases of doubt, the authorities responsible for data protection must be consulted.

5. Ethical guidelines*

When conducting human studies and/or collecting or using human specimens in this project, the recommendations of the Declaration of Helsinki as well as the CIOMS (Council for International Organization of Medical Sciences) and WHO (World Health Organization) "Proposed International Guidelines For Biomedical Research Involving Human Subjects", as amended, must be followed.

6. Ethical approval*

Prior to the commencement of human studies and/or the collection or use of human specimen material, an unqualified positive opinion must be submitted to the competent ethics committee. If the ethics committee does not consider a vote to be necessary, a corresponding declaration from the ethics committee must be submitted.

7. Reservation of withdrawal*

This decision can be revoked and the funding can be discontinued in whole or in part in one of the following cases (reservation of revocation according to § 32 Para. 2 No. 3 in conjunction with § 47 Para. 1 No. 1 SGB X):
in the event that the funding purpose cannot be achieved,

- in cases where funds are blocked for individual estimates of the financing plan,
- in cases where evidence is not submitted within the time limit or is incomplete
- or compelling reasons.

7. Proof of use*

The regulations of No. 14 ANBest-IF apply for the proof of use. The relevant forms will be made available to the recipient of the funding in due course. The form according to Appendix B is to be used for the list of documents.

9. Project events

The sponsor must be informed or invited about important dates or events, newsletters etc. Upon request, information on the type, scope and success of the measures carried out must be provided at any time.

10. Payment of the subsidies

The grants will be paid out at the request of the beneficiary in accordance with No 7 ANBest- IF. Project-related eligible expenses incurred after the start of the grant may be submitted later for settlement with the payment request.

Grants can only be paid out if the funding decision has become final after the deadline for appeals and the confirmation of receipt (form according to Annex E) has been submitted. The grant recipient can make the notice of grant become final before the deadline if he or she waives the right of appeal (form in Annex E).

According to No. 7 Para. 1 Sentence 2 ANBest-IF, the first payment request can be submitted by 31 January 2019. Please note: The payment request must be submitted within four weeks of the start of each quarter for the current quarter.

For the preparation of the payment request, the form according to Annex Z and Annex H must be used. For the status report to be submitted with the payment request (see No. 7 ANBest-IF) the form according to Annex S is to be used.

In addition, each payment request must be accompanied by a report on the current status of case number achievement for the project using the attached form (Annex P).

Consortium partners use the form according to Appendix K to request funds from the consortium management.

11. Refunds
Refunds of grants and, if applicable, interest are to be transferred to the following bank account, specifying the grant code 01VSF18047:

Account holder:
Bank:
IBAN:
BIC:
Gemeinsamer Bundesausschuss Deutsche Apotheker- und
Ärztebank eG DE69 3006 0601 0004 2118 20
DAAEDEDDXXX

12. Commissioning of a project promoter
As project management agency for the Innovation Committee at the Federal Joint Committee, the DLR Project Management Agency is currently responsible for project funding within the framework of the Innovation Fund.

All procedures concerning the implementation and handling of the project must be sent to the project management organization:

DLR Projektträger
– Bereich Gesundheit –
Heinrich-Konen-Str. 1
53227 Bonn

Professional support:
Dr. Sabrina Gollos
Tel.: +49 228 3821-2141
E-Mail: sabrina.gollos@dlr.de

Administrative support:
Daniela Hofmann
Tel.: +49 228 3821-1748
E-Mail: daniela.hofmann@dlr.de

A **copy of the decision** will be sent by e-mail to the project management and the administrative contact person.

With kind regards

Prof. Josef Hecken
This notification was issued electronically and is valid even without an original signature.

Attachments:
- General Subsidiary Provisions of the Innovation Committee at the Federal Joint Committee for Grants from the Innovation Fund (ANBest-IF), as of August 2018: Annex N
- Acknowledgement of receipt and waiver of remedy: Annex E
- financing plan including partial financing plan: Annex F
- Payment request form: Annex Z
- Instructions for completing the payment request: Annex H
- Status report form for the payment request: Annex S
- Form Timetable for achieving the number of cases: Annex P
- Milestone Plan Form: Annex M
- Form of the document list as attachment to the where-used list: Annex B
- Forwarding contract form: Annex W
- Payment request form for consortium partners: Annex K
- G-BA Logo Innovation Committee (only by e-mail): Annex L
- Notes on the use of the G-BA logo Innovation Committee: Annex LH

Information on legal remedies:

An appeal against this funding decision may be lodged within one month of notification with the Berlin-Brandenburg Regional Social Court, Försterweg 2-6, 14482 Potsdam, in writing or for the record of the registrar of the office. The action can also be filed in accordance with the provisions of the Regulation on Electronic Legal Transactions in the State of Brandenburg of 14 June 2006 (GVBl.II/06, [No. 33], p. 558), as amended on 19 December 2017 (GVBl.II/17, [No. 73]), using a qualified electronic signature in electronic form with the electronic mail service of the Berlin-Brandenburg State Social Court via the communication channels specified on the website www.erv.brandenburg.de.
